# Use Patterns, Knowledge Diversity and Drivers for the Cultivation of the Miracle Plant [*Synsepalum dulcificum* (Schumach & Thonn.) Daniell] in Benin and Ghana

**DOI:** 10.3390/plants10112253

**Published:** 2021-10-22

**Authors:** Dèdéou Apocalypse Tchokponhoué, Sognigbé N’Danikou, Nicodème Vodjo Fassinou Hotegni, Daniel Nyadanu, Rémi Kahane, Alfred Oduor Odindo, Enoch Gbènato Achigan-Dako, Julia Sibiya

**Affiliations:** 1 School of Agricultural, Earth and Environmental Sciences, University of KwaZulu-Natal, Private Bag X01, Scottsville, Pietermaritzburg 3209, South Africa; Odindoa@ukzn.ac.za (A.O.O.); Sibiyaj@ukzn.ac.za (J.S.); 2 Laboratory of Genetics, Biotechnology and Seed Science (GBioS), School of Plant Sciences, University of Abomey-Calavi, Abomey-Calavi 01 BP 526, Benin; ndanikou@gmail.com (S.N.); nicodemef@gmail.com (N.V.F.H.); e.adako@gmail.com (E.G.A.-D.); 3 World Vegetable Centre, East and Southern Africa, Duluti, Arusha P.O. Box 10, Tanzania; 4 Ecole d’Horticulture et d’Aménagement des Espaces Verts, Université Nationale d’Agriculture, Kétou BP 43, Benin; 5 Cocoa Research Institute of Ghana (CRIG), Akim Tafo P.O. Box 8, Ghana; dnyadanu@gmail.com; 6 Research Unit HortSys, Department Persyst, CIRAD, Campus de Baillarguet, CEDEX 5, 34398 Montpellier, France; remi.kahane@cirad.fr

**Keywords:** *Richardella dulcifica*, cultural importance, traditional knowledge, NUS, knowledge acquisition, readiness to cultivate

## Abstract

Despite the growing interest in the miracle plant worldwide due to its numerous applications, the threats and the wild harvest of the species hamper its sustainable utilisation. Moreover, traditional knowledge so far documented on the species is limited to a narrow geographical coverage of its natural distribution range, which is West and Central Africa. This study analysed the use variation and knowledge acquisition pattern of the miracle plant among West African sociolinguistic groups and deciphered the drivers of populations’ willingness and readiness to engage in cultivating the species. Semi-structured interviews were conducted with 510 respondents purposively selected from nine sociolinguistic groups in Benin and Ghana using the snowball sampling approach. Information was collected on respondents’ socio-demographic profile, miracle plant ownership, plant parts used and preparation methods, knowledge of the species bioecology, perceived threats on the species, willingness to cultivate, maximum acreage to allocate to the species and maximum price to pay for a seedling. Descriptive statistics, generalized linear models, classification and regression tree models were used for data analysis. The miracle plant ownership mode depended on the age category. Sociolinguistic affiliation, level of schooling, migratory status and religion significantly affected the number of trees owned. We recorded 76 uses belonging to six use categories. The overall use-value of the miracle plant significantly varied according to the respondent sociolinguistic affiliation, main activity and religion. Men were the main source of knowledge and knowledge is mainly acquired along the family line. Knowledge related to food and social uses was mostly acquired from parents and people of the same generation, while magico-therapeutic and medicinal use-related knowledge were inherited from parents and grandparents. Sociolinguistic affiliation, awareness of taboos and market availability were the most important drivers of respondent willingness to cultivate the miracle plant. While the respondent’s level of schooling and perception of plant growth rate determined the maximum acreage they were willing to allocate to the species in cultivation schemes, their main activity, sociolinguistic affiliation and knowledge of the species time to fruiting drove the maximum purchase price they were willing to offer for a seedling of the species. Our findings provide key information for the promotion of miracle plant cultivation in the study area.

## 1. Introduction

The miracle plant *Synsepalum dulcificum* (Schumach & Thonn.) Daniell [Syn: *Richardella dulcifica* (Schumach & Thonn.) Baehni] is a slow-growing West African native tree species belonging to the Sapotaceae family [1]. It naturally thrives in West and Central Africa where it grows on well-drained acidic soils with pH ranging between 4.5. and 5.8 [2]. The species is an intermediate shade-tolerant species [3] that grows well in warm, wet and humid areas. In West Africa (e.g., Benin and Ghana), the species is found in gallery forests, home gardens, backyards, fallows and farms [4,5], and is semi-domesticated, benefiting from weeding, fertilization and pruning as main management practices [5]. *Synsepalum dulcificum* exhibits the highest significance for human wellbeing among the nearly thirty species in the *Synsepalum* genus [6]. The species is well-known for being a unique natural source of “miraculin”, a taste modifying glycoprotein contained in the miracle fruit pulp, which has a sweetening effect on any sour food [7].

The miracle plant has numerous modern and traditional applications in the food and beverage, cosmetics and pharmaceutical industries [2]. In the food and beverage industry, the fruit is used as a non-nutritive natural sweetener and beverage colourant [8], a reliable substitute to synthetic sugar in lemonade and juice [9] and an ingredient in functional yoghurt preparation [10]. In cosmetics, the seed oil is used to prevent hair breakage [11] and improved finger motor skills [12]. It is also utilized in the prevention and treatment of diabetes and cancer, two non-communicable diseases with heavy socio-economic burdens [13,14]. The miracle fruit improves insulin resistance and synthesis [15,16] and restores taste perception in patients undergoing chemotherapy treatment [17]. Consequently, the species could gain expansions of its role in the global health system as projections anticipate 237 million and 6.9 million new cases of diabetes and cancer respectively, by 2045–2050 [18,19]. In addition, high demand for the species in the food and beverage industry is expected due to the increasing pressure and demand for non-nutritive sweeteners. Economically, the miracle fruit has a reeling market value in the USA where a kilogram of the pure powder costs USD 2500 USD (https://www.miraclefruitfarm.com/supplements, accessed on 29 September 2021) and increasing effort is being made for its formal approval in the European Union market [20].

Historically, in West Africa, the miracle fruit has assisted in the consumption of sour foods, unripe fruits and to sweeten local beverages [21], whereas all the non-edible parts of the plant are reported to have nearly 64 medicinal applications (e.g., malaria, tuberculosis and cough treatments) [22]. Despite its occurrence in more than 10 West and Central African countries, the only proper ethnobotanical investigations conducted on the species were limited to some sociolinguistic groups in Benin [22,23] and only sparsely documented information existed on its uses from Nigeria, Togo and Côte d’Ivoire [24,25]. Consequently, there is a paucity of information on the species’ traditional ecological knowledge, that is, the body of knowledge accumulated on the species by local communities through history, by means of direct experience and contact with nature and transmitted from generation to generation [26,27]. The information is necessary to gauge the sustainability of exploitation and contribute to profiling adequate conservation measures [28,29], especially in the centre of origin of the species. Existing reports on the miracle plant reveal that local populations hold a strong knowledge of medicinal applications [23]. Key to the sustainability of these uses is the understanding of the mode of knowledge acquisition and or transmission. However, these have not been documented in the literature.

Agricultural expansion, population growth and overharvesting of plant parts for medicinal purposes are among the threats to the miracle plant in its centre of origin [4,22]. In parallel, current exploitation of the species in West Africa mainly relied on just a few stands in farmers’ backyards, home gardens and farms [5]. Taken together, these two observations suggest that the current supply is unlikely to meet the growing local and international demands for products of the species (e.g., leaves, roots, seeds and fruits) [12,17,20] and to hold its promise as a lever of West Africa economic growth. Cultivation was suggested as a sustainable alternative to semi-wild or wild harvest of plants when demand outgrows natural populations’ capacities [30]. Exploring options for cultivation (as opposed to semi-wild or wild harvest) is therefore desirable in anticipating the predicted increased demand for the potential products that can be derived from the species. For this to be effective, a clear understanding of the factors shaping farmers’ decision making to invest in the cultivation of the species is required. Lessons learnt from previous studies investigating determinants for the cultivation of perennial plants by local populations in West Africa concluded on the prominence of ethnicity, gender, age, instruction level and knowledge of tree biology, as key drivers [30,31,32]. *Synsepalum dulcificum* being of the same nature, these factors are expected to play an important role in farmers’ decision making to cultivate.

The objectives of this study were therefore to analyse the use variation, assess the knowledge acquisition pattern across sociolinguistic groups, gender, level of schooling and age categories and depict factors affecting farmers’ decision to engage in the cultivation of *S. dulcificum*. Specifically, the following research questions were addressed: (i) Do differences in the respondents’ socio-demographic background affect ownership of trees and if so how? (ii) To what extent do the socio-demographic factors affect the species use pattern in West Africa? (iii) What is the knowledge acquisition pattern in the miracle plant, and does it harbour any peculiarities? (iv) What are the most critical drivers of the local population’s willingness and readiness to engage in the cultivation of the miracle plant, and how do they interact with each other?

## 2. Results

### 2.1. Socio-Demographic Characteristics of Respondents

The proportion of women (10%) involved in the study was significantly lower than that of men (90%) (*χ^2^ = 649.6, df = 1*, *p* < 0.001) (Table 1). More than 50% of respondents were 30–59 years old, whereas the young (less than 30-years old) were the least represented. Respondents were on average 55 ± 0.68 years old with a higher proportion of autochthon compared with allochthon (*χ^2^ = 649.6, df = 1*, *p* < 0.001), and most of them did not school with farming as the main activity. Most of the respondents were Christians or practised indigenous religions (e.g., the Thron deity in Benin).

### 2.2. Synsepalum dulcificum Nomenclature, Ownership Pattern and Bioecology

The miracle plant is referred to as Sinssi by the *Adja*; Sislè by the *Aizo*, *Fon* and *Sahouè;* Agbanyun by the *Holli*; Sièsiè by the *Wémé* (Benin); Etimea by the *Akan*; Eliku by the *Ewe*, and Atanmanmi by the *Ga-adangbe* (Ghana). Several other name variants and their meanings were recorded in the two countries (Table 2).

A total of 366 respondents (nearly 72%) owned miracle plant trees. Four ownership modes were recorded including (i) legacy where the respondent inherited the trees from his/her father or grandfather, (ii) self-established where the trees were planted by the respondent himself/herself, (iii) the combination of legacy and self-established and (iv) the spontaneous establishment where the species emerged on the respondents’ land likely following natural dispersion. Nearly 50% of the respondents inherited the trees (*χ^2^ = 88.427, df = 3,* *p* < 0.0001), while self-establishment of trees was recorded in 40% of respondents. A very significant dependence was observed between the miracle plant ownership mode and the age category (*p = 0.004*), the sociolinguistic membership (*p = 0.008*) and the migratory status (*p* = 0.002) of the respondent. While the elderly respondents generally planted the miracle plant themselves, adults and youth mainly inherited them. Likewise, *Akan* and *Ga-adangbe* mostly established their own trees, whereas respondents in other sociolinguistic groups mainly inherited them. Allochthons mainly planted the trees, while autochthons inherited them. No association was observed between the miracle plant ownership mode and gender, level of schooling, or main activity (*p* > 0.05).

**Figure 1:**
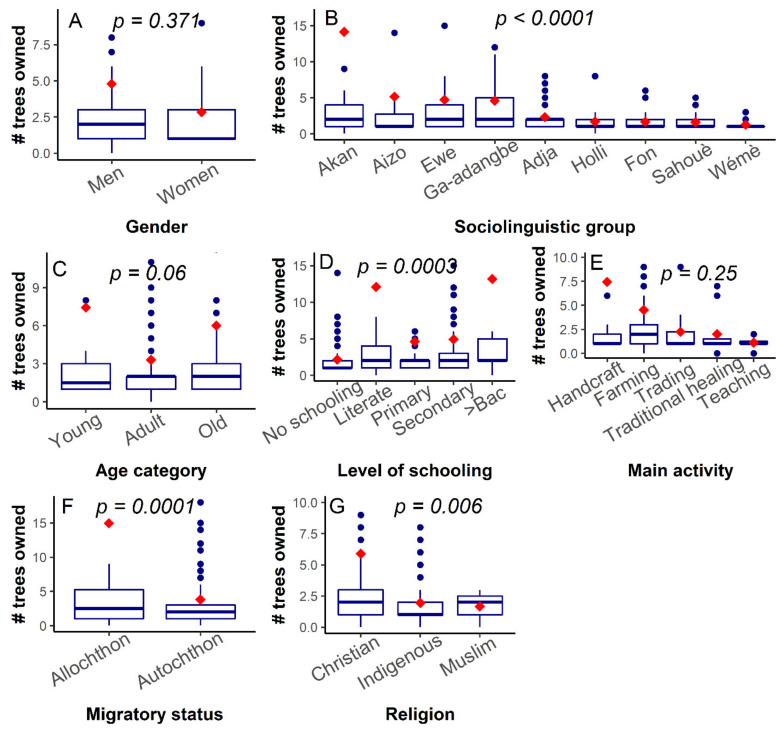
Variation of the number of miracle plant (*Synsepalum dulcificum*) trees owned following various socio-demographic factors in Benin and Ghana. Median values are in bold, red diamond shapes represent mean values, dots above and below boxplots are outliers, and lower and upper tails represent minimum and maximum values, respectively.

The Poisson-modelled generalised linear model indicated that the number of miracle trees owned was significantly affected by the respondent’s sociolinguistic affiliation, migratory status, level of schooling and religion (Figure 1). For instance, the *Akan* held nearly seven-fold more trees (14.15 ± 4.42 trees) than the *Holli* (1.68 ± 0.38 trees) (Figure 1B, *t-value* *= 4.24, p* < 0.0001). Similarly, respondents with a high level of schooling tended to own more trees than those with a lower level of schooling (Figure 1D, *t = −3.62, p = 0.0003*), while allochthons had on average more miracle plant trees than autochthons (Figure 1F, *t* = −3.90*, p* = 0.0001). Christians also owned more trees than Muslim and Indigenous religious practitioners (Figure 1G*, t =* −2.76*, p* = 0.006). Age category only exerted a marginal effect on the number of trees owned (Figure 1C*, t = 1.24, p* = 0.06), whereas neither the gender (Figure 1A) nor the main activity (Figure 1E) significantly affected the number of trees held by the respondents (*p* > 0.05).

**Figure 2:**
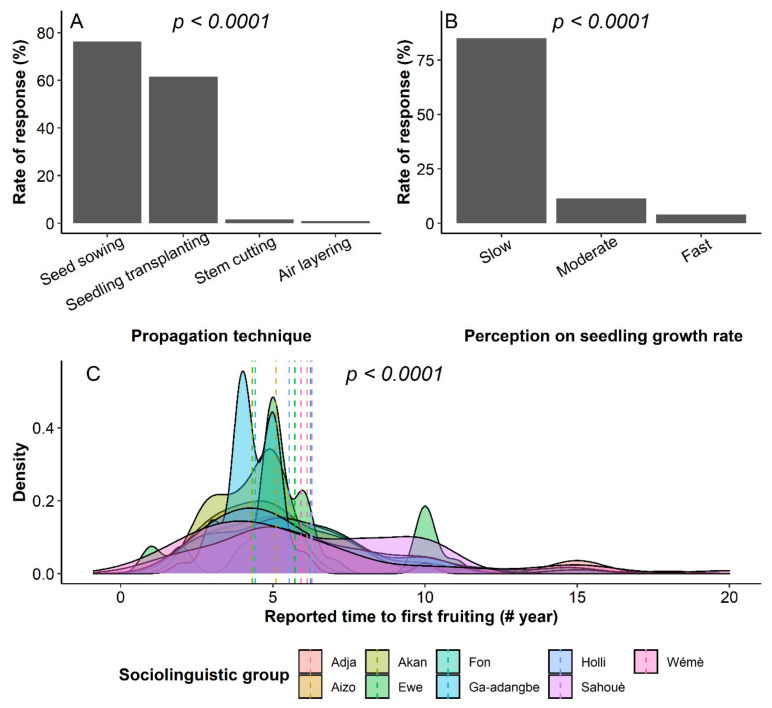
Respondent’s knowledge of the miracle plant (*Synsepalum dulcificum*) regeneration, growth and development in Benin and Ghana. (**A**) Reported propagation techniques employed in the species propagation; (**B**) Perception on the species growth pace and (**C**) Reported time to first fruiting by respondents according to sociolinguistic groups (Each coloured density curve represents the distribution of times to first fruiting of the miracle plant tree within a specific sociolinguistic group, and coloured dotted lines represent the average time to first fruiting within each sociolinguistic group).

The miracle trees were recorded in two types of habitats, namely home gardens and farms. The species main habitat was significantly associated to respondents’ sociolinguistic affiliation (*χ^2^ = 61.26, df = 8, p* < 0.004) with *Adja*, *Akan*, *Ewe* and *Sahouè* mainly having the trees on farms while *Aizo, Fon, Ga-adangbe*, *Wémè* and *Holli* had them in home gardens, though the species was more frequently found on farms (54%) than in home gardens (46%) (*χ^2^ = 3.9696, df = 1, p = 0.04*). Four possible propagation techniques were listed by the respondents, but direct seed sowing and seedling transplanting were the most popular ones (Figure 2A). Most of the respondents indicated that the miracle plant is a slow-growing species (Figure 2B) due to the time to first fruiting averaging 5.5 years. *Akan* and *Ga-Adangbe* reported a time to first fruiting that was significantly shorter than other groups (Figure 2C).

### 2.3. Use Patterns and Knowledge Acquisition in Synsepalum dulcificum

**Figure 3:**
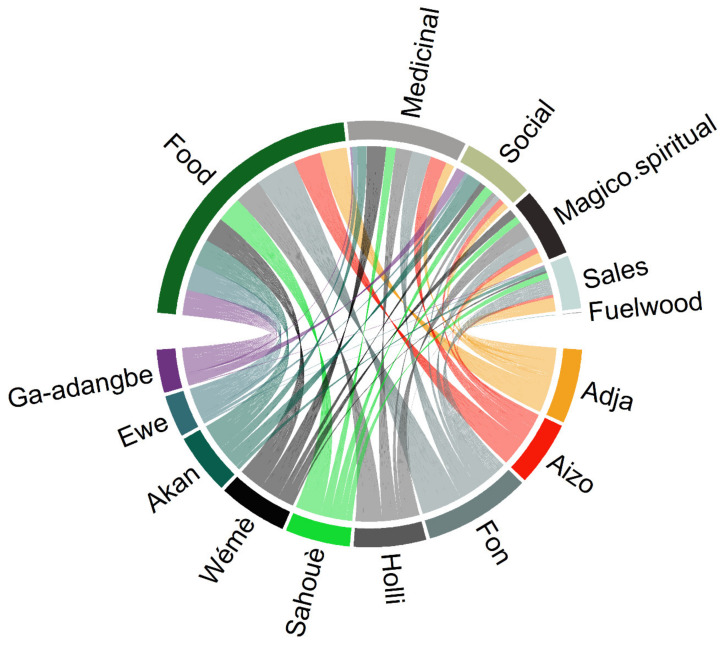
Association between sociolinguistic groups and use category in the miracle plant (*Synsepalum dulcificum*) in Benin and Ghana. Each coloured line joining a specific sociolinguistic group and a use category represent one use report.

Seventy-six uses grouped into six categories namely food, medicines, magico-spiritual, sales, social and firewood were recorded for the species (Table 3). The “medicinal” use category had 56 different uses followed by the “magico-spiritual” use category with 11 uses. A single-use type represented each of the food and fuelwood categories. Magico-spiritual uses were only reported in Benin, whereas the use for firewood was reported only by the *Ewe* in Ghana (Figure 3).

**Figure 4:**
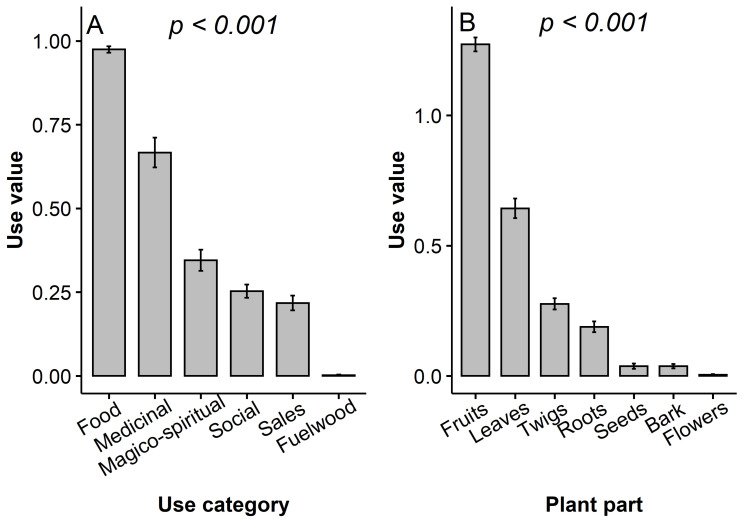
Use-value of *Synsepalum dulcificum* per use category and plant parts in Benin and Ghana. Barsrepresent average use values and error bars represent standard errors or means (SEM).

**Figure 5:**
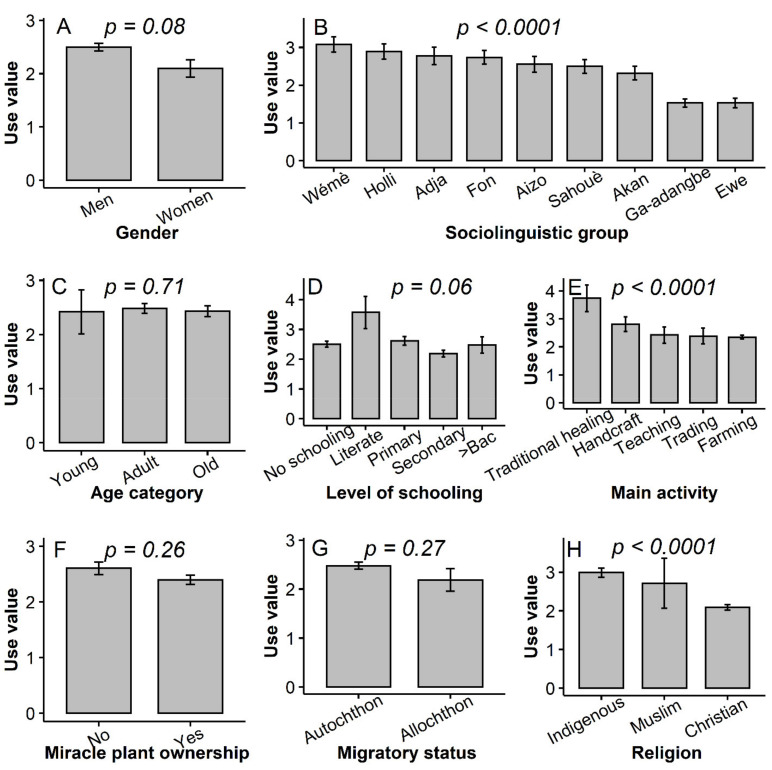
Variation of the miracle plant (*Synsepalum dulcificum*) use-value following various socio-demographic variables in Benin and Ghana. Barsrepresent average use values and error bars represent standard errors of means (SEM).

The overall use-value of the miracle plant was UV = 2.45 ± 0.06. Its disaggregation per use category revealed that the use-value of the food category was significantly greater than that of other use categories (Figure 4A). Likewise, the fruit had a greater UV compared with all other plant parts (Figure 4B). The species UV varied significantly across the sociolinguistic group, the main activity and the religion (*p* < 0.0001) and only marginally following the gender (*p* = 0.08) and the level of schooling (*p* = 0.06) (Figure 5A-H). Sociolinguistic groups in Benin used the species more than sociolinguistic groups in Ghana. The *Wémé* in Benin exploited the species better than any other sociolinguistic groups (Figure 5B). Likewise, the species use value was greater for traditional healers (Figure 5E) and indigenous religious practitioners (Figure 5H) than for respondents in other socio-professional categories or practising other religions. Conversely, no significant effect of age category, migratory status and species ownership status (Figure 5C,F,G) was detected on the use-value (*p* > 0.05). The correlation between use-value and number of trees was overall weak and non-significant (*rho = −0.01*, *p* = 0.89), but differed significantly among attributes of all the studied socio-demographic factors (see Figure S1 and Tables S1–S5). For instance, the lowest correlation between use-value and number of trees owned for the sociolinguistic affiliation was obtained for the *Ewe* (*r = −0.04*, *p = 0.74*), whereas the highest one was observed within the *Akan* community (*r = 0.54, p* < 0.0001) (*Z = 4.86*, *p* = 0.0001). Similarly, the relationship between use-value and number of trees owned was stronger for respondents with a primary level of schooling (*r = 0.3, p* = 0.005) than for respondents with any other level of schooling (*r < 0.08*, *p* > 0.37) (*Z = 3.66*, *p* = 0.01) on one hand, and greater for allochthons (*r = 0.61, p* = 0.002) compared with autochthons (*r = −0.001, p* = 0.98) (*Z = 0.97, p* < 0.0001) on the other hand. Conversely, this association was extremely low for both men and women (*r < 001*, *p* > 0.05) (*Z = 0.97*, *p* = 0.9).

All the miracle plant parts including flowers, fruits, seeds, leaves, twigs, bark and roots are employed in medicinal use with 16 various body systems treated (Table 3). The top five, most frequently treated ailments, in order of importance included: (i) the digestive system, mainly stomach ache and tooth pain; (ii) the general health system, dominated by malaria, fever and asthenia; (iii) the male genital system to stimulate erectile functions and treat male impotency; (iv) the urinary system, mainly enuresis and haematuria; and (v) the circulatory systems, commonly high blood pressure and haemorrhoids. Apart from the bark, all the other plant parts also had magico-spiritual applications, predominantly to bring luck.

The highest consensus among respondents was around the use of the fruit as a sweetener (IAR = 1) and the leaves in blood system ailment treatments (e.g., anaemia) (IAR = 1). These were followed by the use of twigs as chewing sticks (IAR = 0.97), treatment of urinary system ailments (e.g., enuresis) (IAR = 0.96) and in the treatment of general health and digestive systems disorders (e.g., malaria and stomach ache) (IAR = 0.94) (Table 3).

For both medicinal and magico-spiritual uses, the plant parts are employed either fresh/raw or dry and with various methods of preparations (see Figure S2). Decoction, grinding and direct use were the main preparation methods employed when using the plant parts. When used for medicinal purposes the preparations were administered orally, whereas the powder obtained from the grinding of plant parts was used as lapping powder or applied to skin scars followed by incantations in magico-spiritual utilisations. For the preparation, the miracle plant parts were used either alone or associated with other elements or other plant species. Table 4 details some key medicinal and magico-spiritual uses of the species, the preparation modes and the dosage.

**Figure 6:**
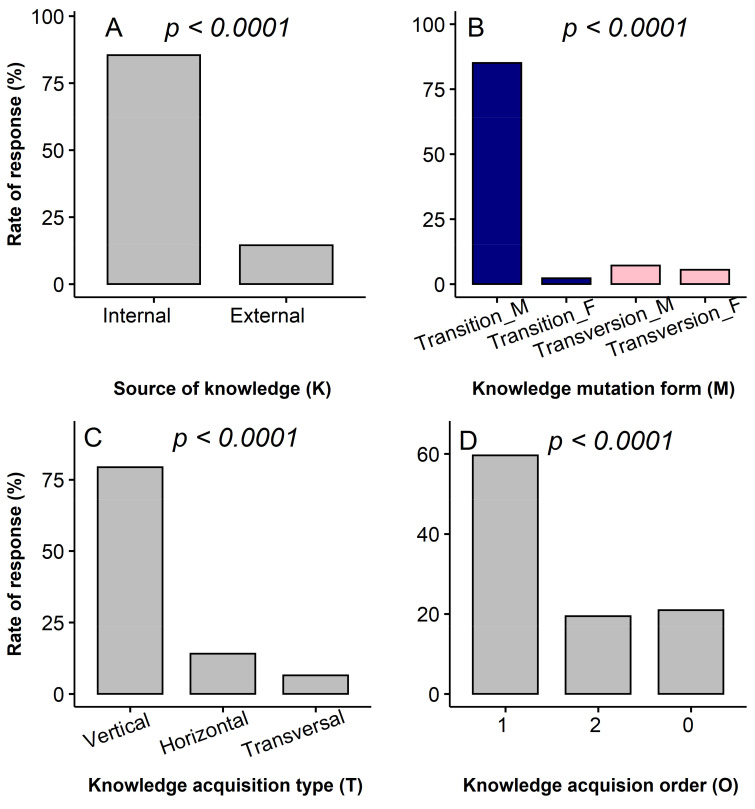
Relative importance of the knowledge acquisition components following the Kernel-Mutation-Type-Order (KMTO) analysis framework in the miracle plant (*Synsepalum dulcificum*) in Benin and Ghana.

**Figure 7:**
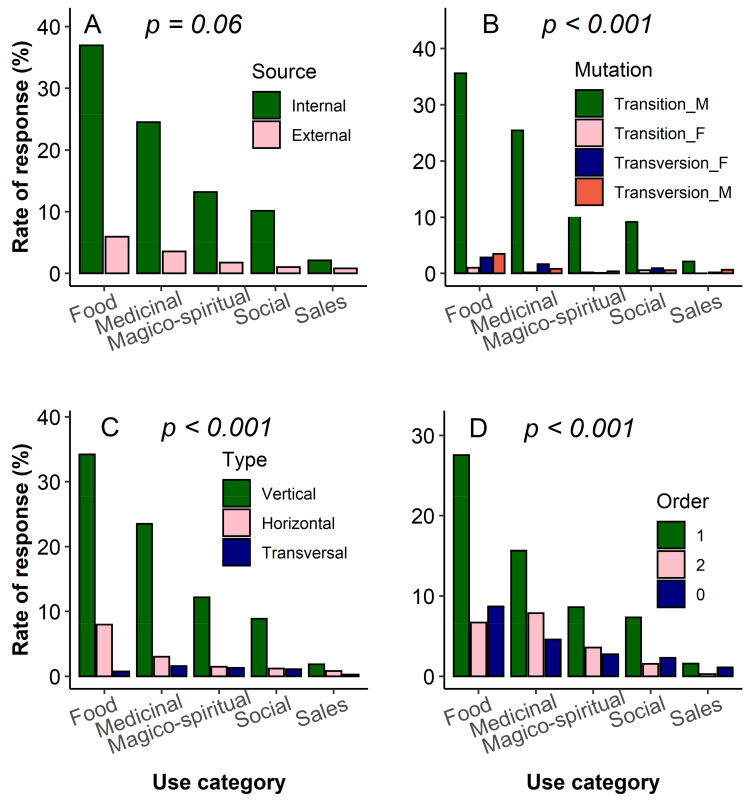
Association between use category and knowledge acquisition path based on the KMTO framework in the miracle plant (*Synsepalum dulcificum*) in Benin and Ghana. (**A**): Source of knowledge, (**B**): knowledge mutation form, (**C**): Knowledge acquisition type and (**D**): Knowledge acquisition order.

The analysis of the knowledge acquisition pattern following the KMTO framework revealed that in general respondents acquired knowledge of the miracle plant from an internal source with a first-order transitionally vertical path marked by the prominence of the father as the main source of knowledge (Figure 6). While the knowledge acquisition source was not linked to the use category (Figure 7A, *χ^2^ = 8.72, df = 4, p* = 0.06), the knowledge mutation form (*χ^2^ = 49.13, df = 12, p* < 0.0001), knowledge acquisition type (*χ^2^ = 9.627, df = 8, p* < 0.0001) and prevailing acquisition order (*χ^2^ =10.12, df = 8, p* < 0.0001) (Figure 7B–D) were conversely all dependent on the use category. Besides the men transition that represented the primary knowledge mutation form, women’s knowledge of food, sales and magico-spiritual use was secondarily acquired from men, while knowledge of medicinal and social uses held by men secondarily came from women. More interestingly, knowledge of medicinal and magico-spiritual uses was inherited more from grandparents than from transmitters of the same generation, whereas knowledge related to the food and social uses was obtained more from transmitters of the same generation than from grandparents.

### 2.4. Perception of Threats, Taboos and Superstitions on Synsepalum dulcificum

**Figure 8:**
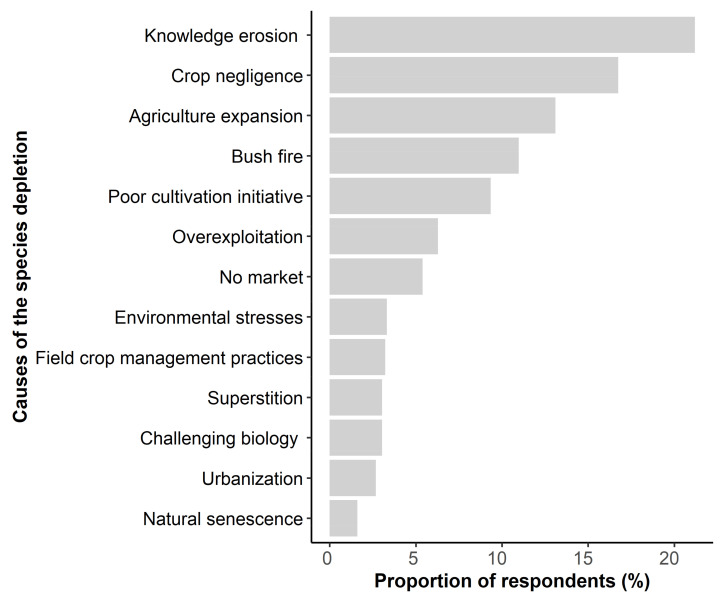
Threatening factors of the miracle plant (*Synsepalum dulcificum*) in Benin and Ghana.

A total of 506 out of the 510 respondents shared their perception of the availability of the species in their environment. Three availability classes: “Decline”, “Stability” and “Increase” were recorded for the species. A significantly higher proportion of respondents (85%) indicated the depletion of the species compared with those indicating stability (6% of respondents) and increase (9% of respondents) (*χ^2^ = 189.3, df = 2*, *p* < 0.001). The perception of the prominence of each availability class depended on the sociolinguistic affiliation (*χ^2^ = 68.052, df = 16, p* < 0.001), the migratory status (*χ^2^ = 15.374, df = 2*, *p* = 0.0004), the religion (*χ^2^ = 26.644, df = 4*, *p* < 0.001) and the level of schooling (*χ^2^ = 31.489, df = 8*, *p* = *0.0001*). For instance, the class “Increase” was predominant over the class “depletion” for Ghana sociolinguistic groups, while the reverse held true for Benin sociolinguistic groups. The major causes of this depletion as reported by the respondents included the erosion of the crop-related knowledge, crop negligence, agricultural expansion, the seasonal bushfires, the lack of cultivation initiatives in the crop and overharvesting (especially of the roots and leaves by medicinal plant vendors) (Figure 8).

Ten taboos and six superstitions were reported in the miracle plant (Table S6). While both taboos and superstitions existed in all sociolinguistic groups sampled in Benin, they were only reported by Ga-adangbe out of the three sociolinguistic groups investigated in Ghana. Additionally, both taboo (*χ^2^ = 23.05, df = 1*, *p* < 0.0001) and superstition (*χ^2^ = 168.3, df = 1*, *p* < 0.0001) were more frequently reported by sociolinguistic groups in Benin than those in Ghana. The commonest taboo on the miracle plant was related to the prohibition to set fire close to the tree. As for the superstition, the most frequently reported one was that “*someone who plants the species will die or one of his parents will die before the plant starts bearing fruits*”.

### 2.5. Determinants of Willingness and Readiness to Cultivate Synsepalum dulcificum

**Figure 9:**
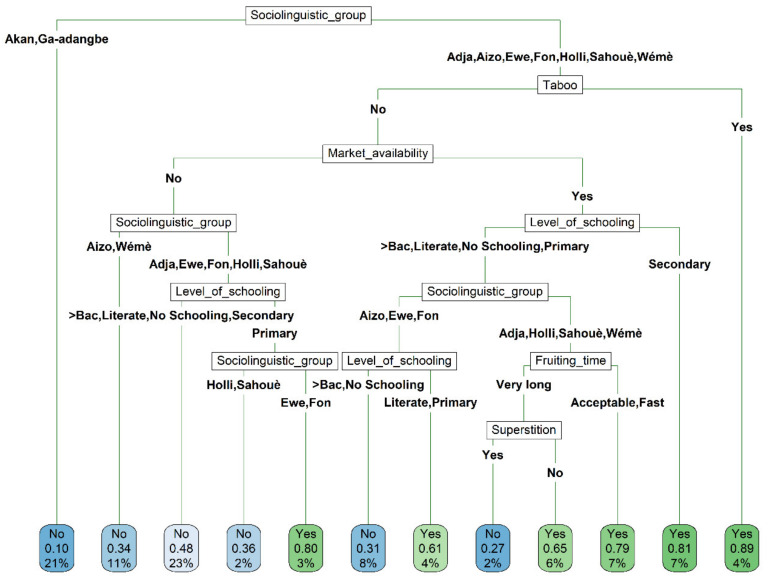
Classification tree depicting the determinants of willingness to cultivate the miracle plant (*Synsepalum dulcificum*) in Benin and Ghana. Each node successively presents the predicted class [willing to cultivate (Yes) or not willing to cultivate (No) the miracle plant tree], the predicted probability of willingness to cultivate and the percentage of observation at the node.

The classification tree model revealed that six out of the 14 candidate variables interacted to significantly determine farmers’ decision to engage in the cultivation of the miracle plant in Benin and Ghana (Figure 9). These variables included the sociolinguistic affiliation, the existence of taboos on the crop, the respondent’s perception of market availability, the level of schooling, the perception of the time to fruiting and the existence of superstition on the crop. While the *Akan* and *Ga-adangbe* were in general not willing to engage in the cultivation of the miracle plant, the decision of their counterparts *Adja*, *Aizo*, *Ewe*, *Fon*, *Holli*, *Sahouè* and *Wémé* was determined by the existence of taboos on the crop, with those reporting taboos more willing to invest in the species cultivation. In absence of taboos, the willingness of *Adja*, *Aizo*, *Ewe*, *Fon*, *Holli*, *Sahouè* and *Wémé* to cultivate the species was conditioned by the interaction between their perception on the existence of a market and their level of schooling. Among those indicating the existence of a market, respondents whose maximum level of schooling was the secondary school level were, in general, more willing to cultivate the species than those who had higher or lower levels of schooling. In this latter group, the *Aizo*, *Ewe*, *Fon* who reached the maximum level of primary school were willing to engage in the species cultivation, whereas the least and most educated *Aizo*, *Ewe*, *Fon* respondents were not willing to do so. For the *Adja*, *Holli*, *Sahouè* and *Wémè* who perceived the availability of a market and who had a level of schooling other than the secondary level, the decision to cultivate the miracle plant was determined by their perception of time to fruiting in the species. Those indicating the species current fruiting time to be acceptable or fast were unsurprisingly willing to engage in the species cultivation, while their counterparts who perceived the time to fruiting as very long accepted to cultivate the species only when there is no superstition on the crop. The *Aizo* and *Wémè* who did not report any known taboos and were not aware of any available market for the species were not ready to cultivate the species. Among the *Adja*, *Ewe*, *Fon, Holli*, *Sahouè* who did not report any taboos, nor perceived any available market for the miracle plant, only the *Ewe* and *Fon* who reached the primary school level were willing to cultivate the miracle plant.

A total of 45% of respondents in this study indicated their willingness to cultivate *S. dulcificum* and the extent of their readiness to do so was evaluated through the maximum acreage they were ready to allocate to the crop and the maximum price they were ready to pay to acquire an ordinary seedling of a miracle plant.

**Figure 10:**
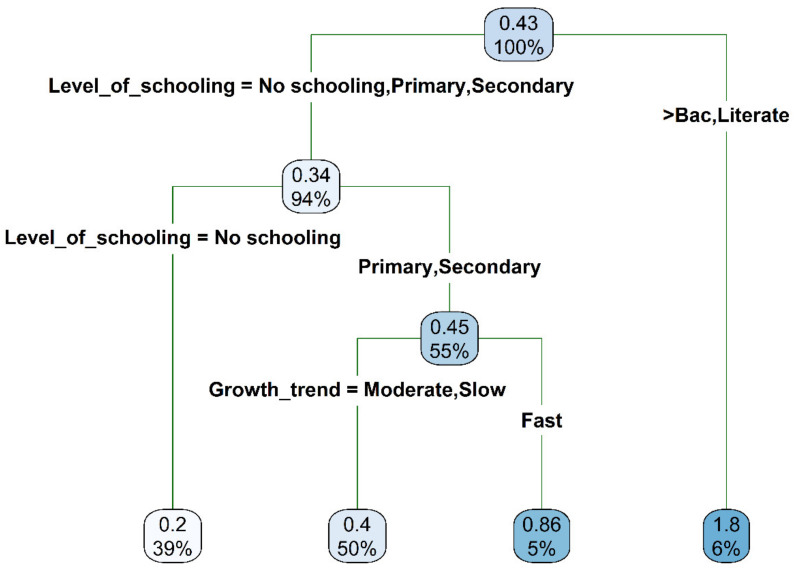
Regression tree depicting the drivers of acreage (ha) allocation as an indicator of respondents’ readiness to cultivate the miracle plant (*Synsepalum dulcificum*) in Benin and Ghana. Each node successively presents the predicted value of the acreage respondents are willing to allocate for the species cultivation and the percentage of observation at the node.

The regression tree model revealed that the only two factors driving plot acreage allocation to the miracle plant by a respondent were by order of importance the respondents’ level of schooling and their perception of the growth rate of the crop (Figure 10). While in general respondents were only ready to allocate on average 0.43 ha of their land for the cultivation of the miracle plant, those reaching the highest level of schooling (University level) were ready to allocate four times more acreage i.e., 1.8 ha to the crop, whereas the respondents who did not school were ready to allocate on average a maximum of 0.2 ha. For the respondents who had intermediate levels of schooling (primary and secondary levels), the acreage to allocate for the cultivation of the miracle plant was conditioned by their perception of the species growth rate. Intermediate levels-educated respondents who perceived a poor growth rate (slow and moderate growth rate) in the species were ready to allocate on average 0.4 ha to the crop, versus two times more acreage i.e., 0.83 ha for their counterparts who perceived the species growth rate as fast.

**Figure 11:**
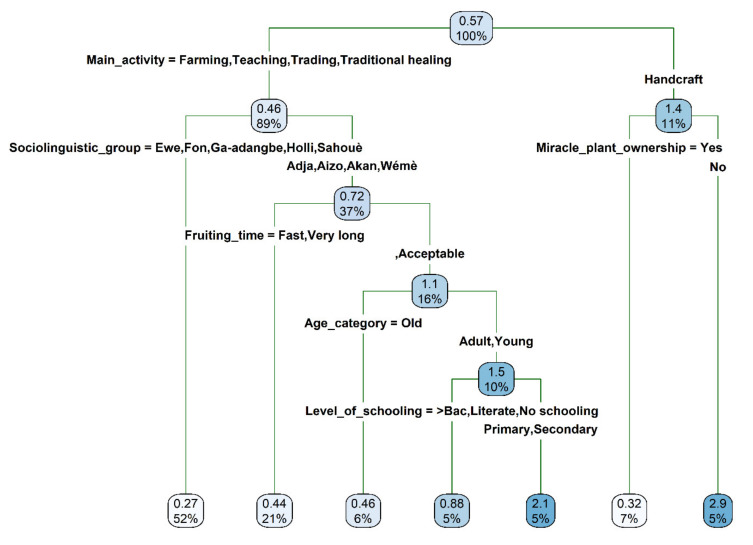
Regression tree depicting the drivers of the maximum purchase price (USD) of a miracle plant (*Synsepalum dulcificum*) seedling as a measure of respondents’ readiness to cultivate the species in Benin and Ghana. Each node successively presents the predicted value of the acreage respondents are willing to allocate for the species cultivation and the percentage of observation at the node.

The miracle plant seedling purchase price as proposed by respondents varied on average from USD 0.27 to USD 2.9 and was mainly driven by the respondents’ main activity, the current miracle plant ownership status of the respondent, the sociolinguistic affiliation, the perception on the time to fruiting, the respondents’ age and level of schooling (Figure 11). While respondents were in general willing to pay on average the amount of USD 0.57 to acquire a seedling of the miracle plant, handcraft makers were willing to pay nearly three times higher price for a seedling and five times higher price when they did not previously own any miracle plants. Conversely, having previously owned a miracle tree decreased to an average of USD 0.4 the amount at which handcraft makers were ready to purchase a seedling of the crop. In contrast to the handcraft makers, the farmers, traders, traditional healers and teachers were only willing to pay an average of USD 0.46 for a seedling of the miracle plant. However, the young and adult *Adja, Aizo*, *Akan* and *Wémé,* who perceived the time to fruiting of the miracle plant as acceptable and who had an intermediate level of schooling (primary and secondary) were willing to pay up to USD 2.1 to acquire one seedling. In contrast, extreme levels of schooling decreased to USD 0.88 the amount this same group of respondents were ready to pay to acquire a miracle plant seedling. As far as the older respondents were concerned, they were only willing to pay an average of 0.4l USD for one seedling.

## 3. Discussion

### 3.1. Synsepalum dulcificum Nomenclature, Ownership Pattern and Bioecology

Folk names used by the local population to designate plant species often reflect a diversity of attributes that may relate to the plant’s habitats, uses, morphology and biological characteristics, among others [28,33,34,35]. The local names recorded for the miracle plant (Table 2) illustrated not only the tree anatomy and longevity but also the functional attributes. Previous ethnobotanical studies suggested that the commonly used names for the miracle plant in Benin reflected the sweetening activity of the fruit [22]. Additionally, the meaning “*No other taste than sweet in contact of the tongue*” (Table 2) given by the *Fon* perfectly illustrated the mechanism of action of the “miraculin”, which is the glycoprotein of the fruit responsible for the sweetening activity. One of the scientifically postulated mechanisms of action of the miraculin is that it binds to the membrane surface of the tongue’s taste cells to trigger the sweetness perception [36]. Thus, sweetness is only perceived when the fruit is in contact with the tongue receptors. The name “Agbanyun” used by the *Holli*, which literally means “honey calabash” was to claim that the miracle fruit holds the paroxysm of sweetness and this strongly aligned with the scientific evidence suggesting the miracle fruit to be 400,000 times sweeter than sucrose [7]. A proper study of plant folk names then holds the potential to help discover detailed plant functions.

Most of the miracle plant trees owned by the respondents were inherited. This strengthened observations by Fandohan et al. [22] in Benin on the species, and confirmed the legacy as an important ownership mode in semi-domesticated plant species [31]. Nevertheless, we observed in this study an influence of the respondents’ age on the predominant ownership mode whereby young and adults representing 63% of respondents (Table 1) inherited more of the species, while the elderly respondents (37% of respondents, Table 1) mainly planted the tree themselves. This prominence of inheritance currently translated an overall poor active conservation of the species and thus raised concerns regarding the future of the species in West Africa since the “older generation” is passing.

Although women held on average three miracle plant trees and men on average five (Figure 1A) this difference was not significant; however, it is concurrent with previous findings which have reported an absence of variation in the total number of miracle plant trees held by men and women in Benin [22]. This similarity in the number of trees owned might partly be attributed to the fact that both women and men are increasingly being exposed to similar social beliefs and norms (e.g., superstition). Because socio-cultural norms strongly differed among sociolinguistic groups and that the perception of such norms and beliefs also varied among religions [37], the observed significant difference among sociolinguistic groups and among religions for the number of miracle plant owned did not come as a surprise [22]. Indeed, the sociolinguistic groups from Ghana practising Christianity at more than 98% (Table 1) held by far more miracle plant trees than Benin sociolinguistic groups whose respondents mainly practised indigenous religions. Because indigenous religions are demarcated from Christianity through the prominence of socio-cultural beliefs and norms, we suspect that these norms (e.g., superstition) likely preclude either form of miracle plant ownership. This hypothesis is furthermore strengthened by the fact that superstitions, which mostly existed in indigenous religions, in Benin in particular, was confirmed as one of the threats factors for the species. An example of such superstition is that “*Someone who plants a miracle plant will die or one of his parents will die before the tree starts bearing fruits*”. This particular superstition was also reported in Eastern Africa on tamarind (*Tamarindus indica*) [38] and sheds light on why the young and adults have not planted the trees themselves. Our results on the species ownership by sociolinguistic groups revealed that the *Holli* held between one and eight miracle plant trees, which is new since no previous studies reported miracle plant trees ownership by this community [22,23].

The miracle plant trees were mainly found in home gardens and on farms in this study. This aligned with findings of previous investigations reporting gallery forests, cultivated farms, home gardens and fallows as possible miracle plant habitats [4,22,23]. Our findings also expand our knowledge of the species habitat in West Africa by highlighting that while home-gardens persist as the major habitat of the species in most of Benin’s sociolinguistic groups, cultivated farms predominated in Ghana.

Although the respondents for the miracle plant currently employ four different propagation techniques, only those based on the seed are the most utilized (Figure 2A). This could be ascribed to the easiness of the transplanting action and the high germination rate of the seeds [1], contrarily to the cuttings that exhibited a recalcitrant adventitious rooting [39]. Interestingly, more than 80% of the respondents perceived the miracle plant as a slow-growing species (Figure 2B), a knowledge that aligned with the experimental conclusions of Tchokponhoué et al. [1] in the species. Similarly, the overall five years (Figure 2C) reported for the age to first fruiting fitted in the time frame of 4–7 years reported for the time to fruiting in natural conditions [40].

### 3.2. Use Patterns and Knowledge Acquisition in Synsepalum dulcificum

Our study expanded the local applications of the miracle plant from 64 uses [22] to 76 uses. Therefore, through our study, miracle plant showed more documented uses compared with marula (*Sclerocarya birrea*, 20 uses) [34], baobab (*Adansonia digitata* L., 38 uses) [41], sweet detar (*Detarium microcarpum* Guill. & Perr., 42 uses) [28] and the red kapok tree (*Bombax costatum* Pellegr. & Vuillet, 46 uses) [42]. The analysis of the importance of each use category suggested that the miracle plant is more medicinal than a food multipurpose species. Though the miracle plant medicinal value was previously indicated [22], this study has updated the medicinal uses and categorized them into body systems (Table 3), making easier and more reliable comparisons with other species. The 16 body systems recorded for the miracle plant alone quantitatively compare well to the number of body systems covered by a set of 309 medicinal plants studied by Ribeiro et al. [43] in Brazil. Likewise, the miracle plant covered more body systems than did a set of 105 medicinal taxa studied in Portugal [44]. These illustrated how deep the medicinal breadth of the miracle plant is and calls for more pharmacological and pharmacognostic investigations to set the scientific bases of the numerous folk utilisations of the species. Good illustrations in this vein exist in Obafemi et al. [16] and Obafemi et al. [45] whose findings enlightened, for instance, the use of the miracle plant leaves by the local population in the treatment of diabetes. Conclusions of many studies indicated that the commonly treated disorders by typical medicinal plants were related to the digestive, circulatory and respiratory body systems [43,46], a trend that is confirmed in this study with the digestive system that was the most treated body system by the miracle plant; then followed by the urinary and circulatory systems. The three most important use categories recorded are concurrent with the findings of Fandohan et al. [22] on the miracle plant and of Assogba et al. [42] on the red kapok tree.

Contrarily to the “age, gender and dynamics of knowledge” hypothesis, which suggested that age category or gender affects an individual’s plant knowledge [47,48] and which was confirmed in a number of species (e.g., *D. microcarpum* [28], *T. indica* [38], *Borassus aethiopum* Mart. [49]), we observed that the miracle plant exhibited similar use-values for both men and women on one hand, and for young, adult and older respondents on the other hand (Figure 5A,C). The absence of differences of use-value among the age categories could indicate that an effective knowledge transmission system from the elderly people to the young is in place in the case of the miracle plant, while the low number of women involved in this study compared to men could partly explain the similar use-value observed between men and women. Alternatively, the comparative advantage that women could have over men due to the medicinal plant status of the miracle plant [50] could have been buffered by the concomitant prominence of magico-spiritual use in the species, a use category mainly reported by men. Conversely, we observed a significant variation of the use-value among sociolinguistic groups (Figure 5B), which concurred not only with previous findings in the species [22] but also with the trend in many other species such as the African locust bean *Parkia biglobosa* (Jacq.) G.Don [46], *A. digitata* [41], *D. microcarpum* [28] and *Gardenia erubescens* tapf & Hutch. [51]. The low use-values recorded for the species in the Ghana sociolinguistic groups versus the high use values obtained in Benin sociolinguistic groups could have arisen from the discrepancy of use categories among countries. Indeed, the “Magico-spiritual use category” that counted 11 different uses was for instance only recorded within sociolinguistic groups in Benin (Figure 3).

Because sociolinguistic groups in Ghana owned more trees than those in Benin (Figure 1B), one could have in the light of the ‘availability hypothesis’ predicted that sociolinguistic groups in Ghana will use the species more than their counterpart in Benin. However, there was a negative and very weak correlation between the use-value and the number of trees owned, which may indicate that the hypothesis does not work for the miracle plant. However, the same availability hypothesis’ also helps predict that because the respondents having the miracle plant in their home garden are closer to the resources than their counterparts holding the species in their farms, they will consequently better use it. In this study, the miracle plant was more used when it was found in home gardens (UV = 2.59) than when cultivated on farms (UV = 2.22). Furthermore, a negative and significant correlation was observed between the use-value and distance of respondent’s dwelling to the trees, thus validating the hypothesis in this latter case. This dichotomy of conclusion strengthened the recommendations of Gaoue et al. [52] to conceptualize the meaning of “availability” when it comes to testing the “availability hypothesis”. The greater use-value of the miracle plant for the traditional healers compared with the other socio-professional categories came without surprise since the species strongly served for both medicinal and magico-spiritual uses, two fields in which the traditional healers are reputed having broader knowledge than any other socio-professional categories [53]. The same also applies to the indigenous religion practitioners, who, being more involved than Muslims and Christians in magico-spiritual events and medicinal uses valued the miracle plant more. As far as the level of schooling was concerned, being highly educated provides less opportunity to be in contact with nature [30] and hence narrows traditional use-related knowledge. This may explain the difference in use-value obtained between respondents who schooled (UV = 2.42) and those who did not(UV = 3.03).

We analysed the knowledge acquisition pattern in this study by employing a framework that helped depict the complex interaction between the transmitters and the receivers from a pedigree and a gender point of view. As previously reported in many species studied in Africa and worldwide [54,55], knowledge acquisition in the miracle plant also occurred mainly within the family network (Figure 6) and orally, which overall means a poor knowledge flow among families within communities and an inefficient acquisition /transmission system since unwritten knowledge is prone to transmission bias due to interpretability. This paucity of knowledge exchange seemed to be particularly marked when it comes to sophisticated knowledge such as medicinal or magico-spiritual uses and this partly helped understand why the huge medicinal use reports in the species were not systematically translated into a high medicinal use-value. Indeed, medicinal and magico-spiritual uses are of high value and then preferably kept secret within families [55]. This prominence of knowledge sharing within the family source unsurprisingly supported the dominance of the vertical knowledge acquisition type observed in this study [54]. In terms of knowledge mutation, knowledge retained by fathers was preferentially passed to sons, but also knowledge retained by women was passed onto a higher proportion to sons compared to daughters (Figure 7), probably as a way to sustain the knowledge maintenance within the family line. Indeed, daughters are called to link to other families through marriage and it is likely that once married they will share the knowledge acquired within their family source with their husbands. So, this fear of girls sharing (even unintentionally) their family knowledge—sometimes considered secret—with their husbands’ family precluded parents to transmit detailed or complex knowledge to them [56].

### 3.3. Perception of Threats, Taboos and Superstitions on Synsepalum dulcificum

Although the global assessment by the International Union for Conservation of Nature (IUCN) indicated the miracle plant as a Least Concern species, there has been growing evidence supporting a local depletion of the species in West Africa [4,22,23]; including this study’s findings as most of the respondents confirmed the number of miracle plants stands in their environment has been sharply decreasing. This study reported 13 various threatening factors for the species in West Africa (Figure 8), which included all the six factors previously documented by Fandohan et al. [22]. Unsurprisingly, agricultural expansion and bush fires, which were commonly indicated as threats to biodiversity [30,34,56] were among the most important causes of the miracle plant depletion. The significance of these two causes in the miracle plant is inflated by the low caloric value of the species, which led to its negligence by the population in favour of known staple species, coupled with an overall poor growth rate making it more vulnerable to bush fires. The 56 various medicinal uses recorded in the species (Table 3) mainly targeted the root and the leaves, two crucial organs for the species fitness, but which are overharvested. Such overharvesting inextricably leads to density reduction; delayed growth and reduced seed production with ultimately a likely impact on the species community [57]. The overexploitation of various plant parts might partly explain why a reduced number of miracle plant trees was recorded in Benin where medicinal uses of the species were mostly concentrated. The most important threatening factor as indicated by respondents in this study was the erosion of knowledge of the species, a cause that came second in the study of Fandohan et al. [22]. Out of the 76 uses recorded, only one (the fruit used as a sweetener) was saliently known in the studied population, indicating that the knowledge of the 75 other uses which are mostly medicinal and magico-spiritual in nature were heterogeneously distributed. Because the conservation through use hypothesis pinpointed that people are more likely to protect a plant resource when they draw a substantial benefit from it [58]; the loss of knowledge of the miracle plant values if no sensitization or awareness-raising actions was taken will continue to threaten the survival of the species in West Africa.

Taboos represented informal institutions whereby social norms lead human behaviour and contributed to biodiversity conservation [59]. Colding and Folke [59] distinguished six categories of taboos (segment, temporal, method, life history, specific-species and habitats taboos); and the prohibition to set fire close to the tree, to urinate on the tree or to touch the root are indirect measures to ensure better protection of the species and avoid the extraction of some sensitive plant parts (e.g., roots). All these falls in the Colding and Folke [59]’s life history taboo category. Likewise, the miracle plant bark and roots have medicinal applications and are often harvested using cutlass. Therefore, the prohibition of having cutlass close to species helps indirectly to prevent roots and bark extraction and foster better growth and productivity. All these align with the taboos as a conservation strategy hypothesis, which predicts that certain plant species are protected using taboos handed down by generations [59,60]. However, the perceived threatened status of the species despite these protection measures seemed to suggest that the enforcement of such life-history taboos is not stringent enough to slow down or reverse the pace of erosion in the current knowledge of this species.

### 3.4. Determinants of Willingness and Readiness to Cultivate Synsepalum dulcificum

Scaling up the cultivation of the miracle plant is crucial for diversifying and sustaining the numerous uses and applications of the species. In this study, we employed classification and regressions tree models (Figure 9, Figure 10 and Figure 11) to identify the most important factors that can serve as levers to achieve this objective. Sociolinguistic affiliation was the most important determinant of the respondent’s willingness to cultivate the species. Although this factor was previously indicated as a determinant of Moringa’ cultivation in Benin [32], its importance was to a lesser extent. In this study, the *Akan* and *Ga-adangbe* who least used the miracle plant clearly are not willing to invest in its cultivation. This aligned with the idea that the knowledge of plant use is crucial for cultivation decisions [30], though not sufficient to exclusively condition it, as many other factors are likely to interact with it. Two of these factors namely market availability and level of schooling were also previously indicated as important cultivation drivers in other plant species [30,61]. This study has shown for the first time that taboos, previously pinpointed as a conservation driver, could also act as a key cultivation driver, especially when backgrounds of the species’ uses clearly exist. Indeed, the various taboos reported in this study contributed to reducing pressure (debarking, branches removal) on the miracle plant, thus fostered an optimal growth of the species, which when coupled with a sound knowledge of use importance guaranteed true benefits for the owner. More importantly, a closer look into our data clearly showed a significantly higher use-value of the species for respondents reporting taboos (UV = 3.37) compared with those not reporting any taboos (UV = 2.41) (*p* < 0.003). Because taboos were conceptualized as a conservation strategy [59] and that cultivation also serves this conservation purpose in addition to foster a higher use of the species, it came without surprise that participants reporting taboos were willing to cultivate the species, potentially as a measure to sustain and expand the benefits from it. As for market availability, it serves as a financial incentive that offered the possibility to trade the harvest and then to make a substantial profit. Consequently, all the respondents who perceived the existence of markets for the miracle plant were willing to engage in its cultivation except for those who did not school or had a high level of schooling and those who reported the existence of superstition in the species. The decision of respondents who did not school not to engage in the cultivation even when the market is available might be explained by their lack of technical knowledge to apply sound management practices that ensure successful cultivation and or to access various networks to trade their product. As for respondents who schooled, we speculated that their decision not to cultivate could be linked to a lack of time for farming activities as they are likely involved in other formal employment. While previous studies established that willingness to cultivate tree species increased with the level of schooling [30,61], we rather identified in this study that intermediate-schooled people were the key group to target in promoting the miracle plant cultivation in West Africa. To the best of our knowledge, no study reported superstition in the miracle plant. Most superstitions reported on the crop ultimately predicted uncomfortable situations or early death for anyone engaged in the cultivation of the species. Because the cost opportunity of benefitting financially from the species at the expense of one’s life is very negligible, the existence of superstitions precluded cultivation, unless the respondent is aware of the early fruiting possibility in the species, knowledge that apparently disrupted the belief in the superstition. A close look into the most dreadful superstition —‘*who plants the miracle plant dies or one of his parents died before it bears fruits’—*recorded in this study revealed that it was likely meant by our forefathers to illustrate the late maturing of the miracle plant but has been misinterpreted. This consequently calls for two urgent actions. First, there is a need for awareness creation to explain to people that they will not die because they have planted the miracle plant tree, but just that the species is slow-growing. Second, this calls for breeders to accelerate research to shorten the growth cycle to develop early fruiting varieties. Besides, the miracle plant is a rare species only owned by a low proportion of the local population, we expected the tree ownership to also trigger willingness for cultivation as a measure to sustain current uses and benefits from the species, which is not the case in this study.

If knowledge of factors that influence the decision to cultivate a plant species is important in devising promotion strategies, modelling factors that affect how much effort the population are ready to deploy to achieve the cultivation objective is crucial for tangible actions. Because land and planting material are two important production factors, this study used the maximum acreage respondents were ready to allocate to the miracle plant and the maximum purchase price of a seedling as proxies of the respondents’ cultivation effort. Our findings suggest that level of schooling and knowledge of the species biology are of paramount importance in the population readiness to cultivate the miracle plant. While acreage allocation increased with the level of schooling, the proposed purchase price for the seedling rather decreased as the level of schooling becomes extreme (very high or too low). By default, higher schooling is associated with wealth in West Africa and the fact that highly educated respondents were ready to allocate more acreage is explained by the fact that they owned more land (45.2 ha on average) compared with respondents with intermediate and low levels of schooling (4.20 ha on average). Contrarily to land ownership that represented a past investment, seedling purchase for cultivation rather stands as an actual or future investment, which respondents with extreme levels of schooling are less ready to make. This observation might be explained by the resource-poor status of respondents with a low level of schooling, while in respondents with a high level of schooling, it rather strengthened their previously indicated poor willingness to cultivate the miracle plant. Conversely, respondents with an intermediate level of schooling in addition to being more willing to cultivate the species also translated it well in terms of both past and future investments for species cultivation. Our results also suggested that providing such a group of respondents with for instance fast-growing miracle plant genotypes would induce more than 100% increase in the acreage they will be willing to allocate for the cultivation of the species (See Figure 10). Activity category previously indicated as a factor affecting willingness to cultivate kola and Moringa in Benin [31,32] was also a key determinant of cultivation effort in the miracle plant, with handcraft makers proposing the highest purchase price (USD 1.4) for one seedling. In addition to owning more trees compared with other socio-professional groups, handcraft makers were also the second socio-professional group to use the species most and seek to sustain and expand their current benefits from the species. This might explain why they were ready to pay higher to acquire the miracle plant. For instance, the miracle plant stem (wood) due to its structural quality was reported to be excellent in making agricultural tools [23], serving as roof and wall poles [62] and also suitable for sculpture shaping. Interestingly, those among the handcraft makers who did not own the species were even willing to pay more to acquire a seedling. Conversely, the elderly being unlikely to benefit over a long period of the species cultivation, were unsurprisingly only willing to pay USD 0.46 to acquire the species versus USD 1.5 for the young and adults.

## 4. Materials and Methods

### 4.1. Study Area

**Figure 12:**
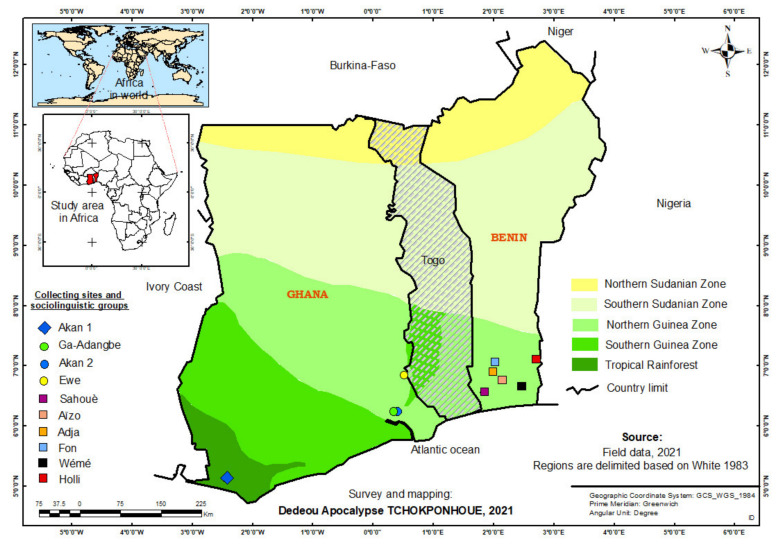
Map showing the study area.

This study was conducted from April to December 2019 in Benin (Latitudes 6°–12°50′ N, Longitudes 1°–3°70′ E) and Ghana (Latitudes 4.7°–11°30′ N, Longitudes 2°50′ W–1°40′ E) (Figure 12), two West African countries known to belong to the centre of origin of the miracle plant. In Benin, the species is confined to the Guineo-Congolian region, in the Northern Guinea zone of West Africa. The region is characterised by a humid tropical climate with an annual rainfall, temperature and relative humidity ranging between 900 and 1300 mm; 25 °C and 29 °C and 69 and 97%, respectively. The dominant vegetation in the region includes savannah, woodland mosaic and relics of dry semi-deciduous forest on ferralitic and ferruginous soils [4,63]. The region is mainly dominated by Kwa- and Yoroboid-speaking groups with more than 10 sociolinguistic groups including Fon, Sahouè, Adja, Aizo, Nagot and Holli, among others [64]. In Ghana, the miracle plant is found in the Transitional and the Deciduous forest [5], shared between the Northern and Southern Guinea ecological zone of West Africa and in the Tropical Rainforest zone of West Africa, which are relatively moister than the Guineo-Congolian zone of Benin. The annual precipitation in these regions ranges from 800 mm to 2800 mm with a mean annual temperature between 26.1–26.4 °C [65]. These regions are dominated by deciduous tropical forests and lush forested vegetation cover, whereas the main soil types include nitosols, acrisols and ferralsols [66]. Major sociolinguistic groups include Akan, Ewe and Ga-adangbe.

### 4.2. Respondents’ Sampling

Nine sociolinguistic groups (six in Benin and three in Ghana, Table 1) that use the species [5,22] were targeted in this study. As we were only interested in the actual use-value of the species, and because the theoretical knowledge might not be systematically translated into actual use value [67], respondents were purposively sampled using the snowball technique. A respondent comprised a person who: (i) was able to identify the species including recognizing the miracle fruit from a photograph (See Figure S3) and knowing its local names, (ii) effectively used at least one part of the miracle plant in his/her lifetime, and (iii) gave his/her prior and informed consent to participate in the study. In total, 510 respondents across the groups, with an average between 51 and 87 per sociolinguistic group were interviewed (Table 1).

### 4.3. Data Collection

Face-to-face semi-structured interviews based on a questionnaire were used for the data collection. A verbal agreement was obtained from traditional authorities of local communities prior to administering the questionnaire. Interviews were conducted in the respondent’s preferred languages, which were either his/her local language, French, or English. Direct interviews using either English or French were done only when the respondents desired so. Otherwise, each interviewer (where necessary) was accompanied by a well-trained local guide (who understood both French/ English and the interviewee-spoken language) to ease the questions/answers translation. The data collected were related to (i) the socio-demographic information (gender, age, sociolinguistic affiliation, religion, migratory status, land ownership, level of schooling and main activity) of the respondents, (ii) the local names of the miracle plant and their meanings, (iii) the miracle plant ownership status, mode of acquisition and number of trees owned, (iv) the plant parts used and use forms, (v) the method of preparation for each indicated use, (vi) the source of knowledge for each use mentioned, (vii) the respondents’ perception of the conservation status of the species and the perceived threatening factors if any, (viii) the knowledge of the species’ bioecology, (ix) the taboos (prohibitions imposed by social custom or as a protective measure [59]) and superstitions (beliefs not based on reason or knowledge [68]) on the species, (x) the respondents’ willingness to engage into cultivating the species, (xi) the maximum acreage the respondent is ready to allocate for the cultivation of the species (when answer to the question (x) is yes) and (xii) the respondent-proposed maximum purchase price of a seedling of the species.

### 4.4. Data Analysis

The analyses were performed in the R environment (V. 3.6.2) [69].

The socio-demographic profile of respondents was summarized using descriptive statistics (mean, standard error of the mean, frequency) computed with the functions *descript ()* and *crosstab ()* of the package “misty” [70]. Content analyses were conducted to assess the species local names variation across sociolinguistic groups. The difference in the preponderance of the recorded miracle plant acquisition modes was tested using a Chi-square multiple proportion comparison test with the function prop. test *()* of base R. Similarly, the dependence between socio-demographic characteristics and the miracle plant acquisition mode was tested using either a Chi-square test or a Fisher’s exact test (to account for contingency table containing values lower than 5) of independence. To test if the number of trees owned were affected by the respondents’ socio-demographic characteristics, we used a Poisson- or quasi-Poisson- (to account for overdispersion) fitted generalised linear model (glm). To assess the respondent’s knowledge of the species bioecology, we first used a Chi-square test to assess the existence of any association between miracle plant habitat and the studied sociolinguistic groups. Second, a multiple proportion comparison test was used to understand the relative importance of different propagation modes reported for the species in the study area. The same test was also used to depict the perception of the respondents on the species growth trend, whereas the difference in the species time to fruiting as indicated by respondents from various sociolinguistic groups was tested using a Poisson-fitted glm. Medicinal uses were classified into body systems following the International Classification of Primary Care 2 (ICPC2) [71] and in use categories. We used the function *ethnoChord ()* of the “EthnobotanyR” package [72] to graphically illustrate the association between sociolinguistic groups and use categories. To assess the extent of species valuation by each respondent, the species use value per respondent (UVj) was computed following Equation (1):

(1) $${\mathrm{UV}}_{j}=\sum_{i=1}^{n}{\mathrm{UR}}_{I}$$

where ${\mathrm{UR}}_{i}$ is a specific use report by the respondent j and n is the total number of use reports mentioned.

From UV_J_, the total use-value of the species was computed afterwards following Phillips and Gentry [73] using Equation (2):

(2) $$\mathrm{UV}=\frac{{\mathrm{UV}}_{j}}{N}\;$$

where N is the total number of respondents.

The variation of UV following the different socio-demographic factors was analysed by fitting a glm with a Poisson/quasi-Poisson-error structure. The use-value and the number of trees owned were correlated using a Spearman/Pearson correlation, while the statistical difference of correlations among attributes of each socio-demographic factor was tested using a Fisher’s R-to-Z transformation. To assess the specificity of plant parts for their use in a particular category or to treat a particular body system, we computed the informant agreement ratio (IAR) following Bakwaye et al. [74] using Equation (3):

(3) $$\mathrm{IAR}\;=\frac{\left({\mathrm{nr}}_{i}-{\mathrm{na}}_{i}\right)}{\left({\mathrm{nr}}_{i}-1\right)}$$

where nr_i_ is the total number of citations recorded for a given use category or body system i and na_i_ is the total number of different plant parts that are employed in this specific use category or body system i.

**Figure 13:**
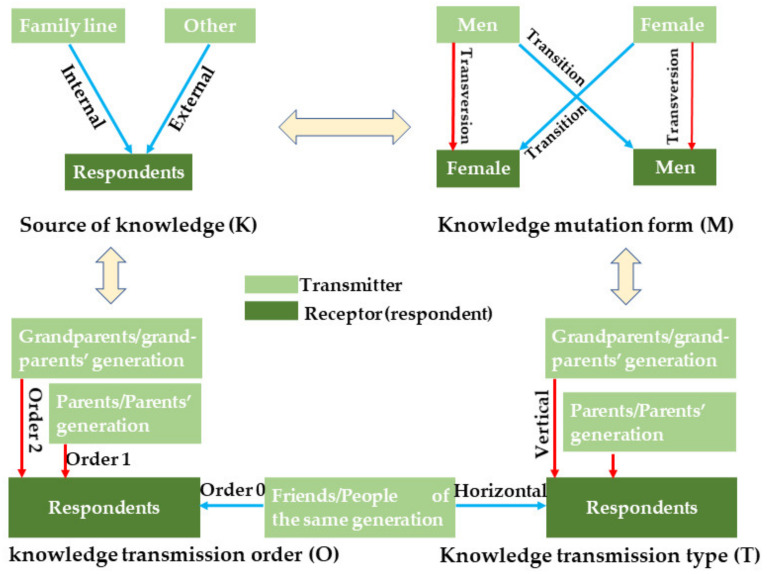
Graphical illustration of the Knowledge Mutation Type Order (KMTO) analysis framework.

To analyse the knowledge acquisition pattern in the species, we developed a framework called “KMTO” that brought together four knowledge attributes including (i) the source or kernel of the knowledge “K”, (ii) the knowledge mutation form “M”, (iii) the knowledge acquisition type “T” and (iv) the knowledge acquisition order “O”. In this model, the source of the knowledge K had two states: “internal” where the respondent acquired the knowledge along his/her family line (e.g., from a nephew, the grandfather, a cousin, etc.) and “external” where the knowledge is acquired from a source other than the respondent’s family (e.g., the community or a friend). The knowledge mutation form “M” is either a “transition” where the knowledge transmitter and the respondent have the same sex (men from men or women from women) or a “transversion” where the transmitter and the respondents were of different sex (men from women or women from men). The acquisition type “T” has three states: “vertical” where the knowledge was acquired from another generation (e.g., respondent acquiring the knowledge from his father or grandmother), “horizontal” where the knowledge was acquired from the same generation (e.g., respondent acquiring the knowledge from a sister or a cousin or a person of the same generation) and “transversal” in which the knowledge was acquired through a self-learning experiment (e.g., knowledge acquired from a book). The knowledge transmission order “O” is defined as either “1” (respondent acquiring the knowledge from a direct progenitor: father or mother), “2” (the respondent acquired the knowledge for the grandfather/ grandmother) or “0” (characteristic of horizontal and transversal knowledge acquisition type). A graphical illustration of this KMTO framework is presented in Figure 13. Following this framework, specific knowledge can be acquired following 36 different paths. We compared the overall importance of each knowledge attribute state using a multiple proportions comparison with the function *prop.test ()*. A Chi-square test or a Fisher.exact test of independence was used to test the association between the predominant state of each knowledge attribute and the species use category.

To identify the drivers of respondents’ decision making to engage or not in the cultivation and depict the interactions among these factors we built a classification tree model using the function *rpart ()* with the “*class*” method in the “rpart” package [75]. When the respondent is favourable for the cultivation, the drivers of cultivation readiness assessed through the maximum acreage to allocate and the maximum price to pay for a seedling were assessed by building a regression tree model, still in the “rpart” package, but with the “*anova*” method this time. Finally, the function *plot.rpart ()* of the “rpart.plot” package [76] was used to visualize trees generated for the classification and regression tree models.

## 5. Conclusions

The present study revealed that the miracle plant is a multi-purpose medicinal plant species with a diversity of local names, mostly known for its food value in Benin and Ghana. The species has a higher cultural importance for sociolinguistic groups in Benin compared with their counterparts in Ghana, with magico-spiritual uses being a key segregating component. Youth and adults were as knowledgeable as elderly respondents were, while traditional healers and indigenous religion practitioners valued the species the most. Men were the main source of knowledge and knowledge is mainly acquired along the family line. Our findings also suggested a heterogenous pattern of knowledge acquisition whereby soft knowledge (e.g., food use and social use) was mostly acquired from parents and people of the same generation, while sophisticated knowledge (e.g., magico-therapeutic and medicinal uses) was inherited from parents and grandparents. The predominant ownership mode of the species supported its overall depleting status, which calls for the necessity to take more active conservation measures to sustain utilization. To that end, we evidenced that socio-cultural, economic and biological factors are key levers to consider when engaging West African (e.g., Benin and Ghana) local populations into the cultivation of the miracle plant. From a cultivation perspective, respondents were overall willing to pay up to 1.9 USD to acquire a seedling and to allocate up to 2.8 ha of their land. These figures were mainly modulated by the sociolinguistic affiliation, the market availability, the instruction level, the knowledge of the species biology, the activity category as well as social beliefs (e.g., taboo and superstition). We established that while taboo could serve as a serious driver for miracle plant cultivation, superstition clearly precludes it, hence the necessity for more investigation of social belief systems in other orphan crops. Our findings represent key decision-making tools to hasten the promotion of the miracle plant in the study area, and in West Africa in general, specifically suggesting respondents with an intermediate level of schooling as key targets to achieve this objective.

## Figures and Tables

**Table 1:** Proportion of the respondents falling within different socio-demographic characteristics in Benin and Ghana.

		Ghana			Benin						
Factor	Modalities	Akan (n = 53)	Ewe (n = 53)	Ga-adangbe (n = 51)	Adja (n = 54)	Aizo (n = 56)	Fon (n = 87)	Holli (n = 55)	Sahouè (n = 50)	Wémé (n = 51)	Total (N = 510)
Gender	Men	84.91	84.91	86.27	92.59	92.86	88.51	98.18	88.00	94.12	90.00
	Women	15.09	15.09	13.73	7.41	7.14	11.49	1.82	12.00	5.80	10.00
Age category	Young: <30 years	5.66	3.78	0.00	7.41	1.79	6.90	56.36	0.00	0.00	9.00
	Adult: 30–59 years	54.72	52.83	56.86	61.11	48.21	60.92	38.18	50.00	58.82	54.00
	Old:≥60 years	39.62	43.39	43.14	31.48	50.00	32.18	5.46	50.00	41.18	37.00
Level of schooling	No-schooling	5.66	3.77	21.57	51.85	51.79	47.13	76.36	50.00	31.37	38.00
	Literate	3.77	0.00	1.96	7.41	0.00	2.30	7.27	0.00	1.96	03.00
	Primary	13.21	16.98	27.45	16.67	25.00	28.73	14.55	24.00	37.25	22.00
	Secondary	67.93	69.81	47.06	24.07	23.21	17.24	1.82	20.00	23.54	33.00
	>Bac *	9.43	9.44	1.96	0.00	0.00	4.60	0.00	6.00	5.88	04.00
Main activity	Farming	84.91	94.34	98.04	85.19	71.43	54.02	87.27	90.00	72.55	82.00
	Handcraft	9.43	3.77	1.96	7.41	10.71	25.29	1.82	2.00	9.80	08.00
	Teaching	1.89	1.89	0.00	0.00	3.57	6.90	0.00	6.00	1.97	03.00
	Trading	3.77	0.00	0.00	3.70	3.57	9.20	0.00	0.00	7.84	03.00
	Traditional healing	0.00	0.00	0.00	3.70	10.72	4.59	10.91	2.00	7.84	04.00
Religion	Christian	98.11	98.11	100.00	38.89	55.36	44.83	7.27	34.00	62.75	60.0
	Indigenous	0.00	0.00	0.00	61.11	44.64	55.17	92.73	66.00	27.45	39.00
	Muslim	1.89	1.89	0.00	0.00	0.00	0.00	0.00	0.00	9.80	01.00
Migratory status	Autochthon	83.02	92.45	94.12	92.59	96.43	87.36	100.00	100.00	92.16	93.00
	Allochthon	16.98	7.55	5.88	7.41	3.57	12.64	0.00	0.00	7.84	07.00

>Bac *: University level.

**Table 2:** Diversity and meaning of the miracle plant (*Synsepalum dulcificum*) nomenclature in Benin. Dominant name (s) in each sociolinguistic group are italicized.

Country	Sociolinguistic Groups	Local Names	Meaning
Ghana	Akan	*Etimea,* Asaaba, Aswan	Something sweet
	Ewe	*Elẽ, Eliku,* Elindidi	Something naturally sweet
	Ga-adangbe	*Atanmanmi*	Something sweet
Benin	Adja	*Sinssi*	-Something sweet
			Something simultaneously sweet and bitter
			Hard and long-living element
	Aizo	*Sièsiè, Sisrè*	Something sweet
	Fon	*Sislè, Sisrè*	A naturally sweet fruit
			Something whose fruits are sweet but the leaves and root are bitter
			The co-spouses
			No other taste than sweet in contact with the tongue
	Holli	*Agbanyun*	Honey’s calabash
	Sahouè	*Sisrè*	Something sweet
	Wémé	*Sièsiè*	Something sweet every time

**Table 3:** Use category, body systems and informant agreement on the documented uses for the miracle plant (*Synsepalum dulcificum*) in Benin and Ghana.

Category	Sub-Category/Body Systems	Use	Number of Use Report	Plant Parts Involved	IAR
Food	Sweetener	Sweetener	497	Fruits	1.00
Sales	Commercialization	Commercialization	112	Fruits, leaves, roots, seeds and twigs	0.96
Social	Handcraft	Chewing stick	117	Twigs	1.00
	Tool	Hoe handle	1	Twigs	NA
	Harmony	Conflict resolution, against alcoholism, miraculin activity disruption, accelerating the walk of the new-born, Improving voice	10	Fruits, leaves, roots, seeds, and twigs	0.55
Magico-spiritual	Lucky charm	Lucky charm	95	Flowers, fruits, leaves, roots and twigs	0.95
	Woe induction	Woe induction	21	Seeds, leaves, fruits and twigs	0.85
	Bewitchment	Bewitchment	15	Leaves, roots, seeds and twigs	0.78
	Protection	Protection	13	Leaves, roots and twigs	0.83
	Love attraction	Attract love	12	Fruits, leaves and twigs	0.81
	Wealth attraction	Wealth attraction	8	Fruits, leaves, roots and twigs	0.57
	Ritualistic	Ritualistic	12	Fruits, leaves and roots	0.81
Medicinal	Blood	Anaemia	3	Leaves	1.00
	Circulatory	Haemorrhoid, heart pain and hypertension	22	Bark, leaves, roots, and seeds	0.86
	Digestive	Absence of taste perception, angina, caries, jaundice, diarrhoea, hepatitis, purgative, sore throat, stomach-ache, tooth decay, tooth pain, ulcer, and vomiting	90	Bark, fruits, leaves, roots, seeds and twigs	0.94
	Endocrine, metabolic, and nutritional	Loss of appetite for food, diabetes, and overweight	18	Fruits, leaves, roots and seeds	0.82
	Eye	Eye pain	2	Fruits and leaves	0.00
	Female genital system	Cyst, menstrual pain, and menstrual irregularity	9	Leaves and roots	0.875
	General health and unspecified	Excessive fatigue, cancer, fainting, fever, malaria, measle	68	Bark, fruits, leaves, roots and seeds	0.94
	Male genital system	Male impotency and manhood stimulation	35	Bark, fruits, leaves, roots, seeds and twigs	0.85
	Musculoskeletal	Back pain, Chest pain and hip-ache	6	Leaves and roots	0.80
	Neurological	Headache and lack of reactivity to stimuli	6	Fruits and leaves	0.80
	Pregnancy and childbearing	Delivery, lactation stimulation, weaning and miscarriage	20	Bark, fruits, leaves and roots	0.84
	Psychological	Lack of sexual appetite of women and Memory-aid	20	Bark, flowers, fruits, leaves and roots	0.78
	Respiratory	Cough, respiratory troubles	10	Bark, fruits, leaves and roots	0.66
	Skin	Wound healing and boil	6	Bark, leaves and roots	0.60
	Urinary	Presence of blood in urine, Enuresis and Kidney ailment	26	Leaves and roots	0.96
Firewood	Firewood	Firewood	1	Branches/twigs	NA*

* NA: Not calculated; only one (1) use report.

**Table 4:** Detailed description for some selected medicinal and magico-spiritual applications of the miracle plant (*Synsepalum dulcificum*) in Benin and Ghana.

Medicinal/Magico-Spiritual Application	Description
Lucky charm for traders and candidates to exams (Benin)	Chewing of the fresh leaves of *S. dulcificum* together with a fruit of *Cola nitida* (Vent.) Schott & Endl. as needed in the morning
	Grinding the miracle plant leaves and use the powder as an ingredient to prepare a soap that will be used every day
	The preparation obtained by grinding together the dry miracle plant roots, leaves and fruits + dry leaves of *Arbrus precatorius* L.+ *Garcinia kola* (Heckel) is shaped in small balls that are kept under the tongue
	Prepare an infusion of the mixture of the leaves of the miracle plant and seeds of *Afromomum melegueta* [Roscoe] K. Schum to which some sugar or honey is added. The infusion is drunk when needed
Haemorrhoid (Benin + Ghana)	Infusion of the root of the miracle plant + other non-disclosed plants + kaolin to be taken when needed
	The decoction of the miracle plant’s leaves together with the bark associated with other non-disclosed plants is taken 3 times per day for a week.
Cough (Benin)	The decoction of the miracle plant bark mixed with the leaves of *Casuarina equisitifolia* L. is drunk daily until the disease stopped
Enuresis (Benin)	Add residues of a hen’s nest to the miracle plant leaves and make a decoction of it that will be drunk as long as needed
Headache (Benin + Ghana)	Dry and grind altogether the leaves of the miracle plant with the seeds of *Afromomum melegueta* [Roscoe] K. Schum. The powder obtained is then topically applied to the skin scars made on the forehead of the sick person.
Ulcer (Benin)	Decoction of the leaves of the miracle plant + leaves of *Heterotis rotundifolia* (Sm.) Jacq-Fél. to be taken when the pain is felt
Stomach-ache (Benin + Ghana)	Pour ground dry seeds of miracle fruit in warm water and drink it. Drink the alcohol-based decoction of the mixture of the miracle fruit, leaves and roots.

## Data Availability

All data generated or analysed during this study are included in this published article (and its Supplementary Materials).

## Supplementary Materials

The following are available online at https://www.mdpi.com/article/10.3390/plants10112253/s1, Figure S1: Regressions lines illustrating the association between use-value and number of trees for attributes of different socio-demographic factors in Benin and Ghana; Figure S2: Relative importance of different preparation methods (all plant parts combined) for medicinal and magico-spiritual applications of the miracle plant (*Synsepalum dulcificum*) in Benin and Ghana; Figure S3: Plate used for miracle plant identification by the selected respondents: A—the fruits (miracle fruits) and B— branch bearing fruits and other plant parts (leaves and twigs) commonly used; Table S1: Fisher R-to-Z test among sociolinguistic groups for the correlations between the number of trees owned and use-value. Values in bold are the correlation coefficients between number of trees owned and use-value; Table S2: Fisher R-to-Z test among activity categories for the correlation between the number of trees owned and use-value. Values in bold are the correlation coefficients between number of trees owned and use value per age category.; Table S3: Fisher R-to-Z test among respondents with different levels of schooling for the correlation between the number of trees owned and use-value. Values in bold are the correlation coefficients between number of trees owned and the use-value for the different instruction levels.; Table S4: Fisher R-to-Z test among respondents in various activity sectors for the correlation between the number of trees owned and use-value. Values in bold are the correlation coefficients between number of trees owned and the use-value.; Table S5: Fisher R-to-Z test among respondents of religions for the correlation between the number of trees owned and use-value. Values in bold are the correlation coefficients between number of trees owned and the use-value.; Table S6: Taboos and superstitions reported in the miracle plant (*Synsepalum dulcificum*) in Benin and Ghana. Taboos and superstitions in bold were recorded in Ghana while those in regular font were recorded in Benin. For Tables S1–S5, values in bold are the correlation coefficients between number of trees owned and the use-value. Values in the lower diagonal are the p-values associated with the Fisher R-to-Z test and those in the upper diagonal are the associated Z statistics.
