# Supplementary material for: Use Patterns, Knowledge Diversity and Drivers for the Cultivation of the Miracle Plant [Synsepalum dulcificum (Schumach & Thonn.) Daniell] in Benin and Ghana
Source: Plants (Basel). 2021 Oct 22;10(11):2253. doi: 10.3390/plants10112253 (PMC8620124; doi:10.3390/plants10112253)
Supplement: Supplementary file 1 [file plants-10-02253-s001.zip › plants-1346032-supplementary.pdf]

Use Patterns, Knowledge Diversity and Drivers for the Cultivation of the Miracle Plant [*Synsepalum dulcificum* (Schumach & Thonn.) Daniell] in Benin and Ghana

Dèdéou Apocalypse Tchokponhoué[1,2,*], Sognigbé N'Danikou[2,3,6], Nicodème Vodjo Fassinou Hotegni[2], Daniel Nyadanu[4], Rémi Kahane[5], Alfred Oduor Odindo[1], Enoch Gbènato Achigan-Dako[2] and Julia Sibiya[1]

[1]School of Agricultural, Earth and Environmental Sciences, University of KwaZulu-Natal, Private Bag X01, Scottsville, 3209, Pietermaritzburg, South Africa; Odindoa@ukzn.ac.za (A.O.O.); Sibiyaj@ukzn.ac.za (J.S.)

[2]Laboratory of Genetics, Biotechnology and Seed Science (GBioS), School of Plant Sciences, University of Abomey-Calavi, 01 BP 526, Abomey-Calavi, Republic of Benin; ndanikou@gmail.com (S.N.); nicodemef@gmail.com (N. V.F-H.); e.adako@gmail.com (E.A.D.)

[3]World Vegetable Centre, East and Southern Africa, Po. Box 10 Duluti, Arusha, Tanzania.

[4]Cocoa Research Institute of Ghana (CRIG), P. O. Box 8, Akim Tafo, Ghana; dnyadanu@gmail.com

[5]Research Unit HortSys, Department Persyst, CIRAD, Campus de Baillarguet, 34398 Montpellier

Cedex 5, France; remi.kahane@cirad.fr

[6]Ecole d'Horticulture et d'Aménagement des Espaces Verts, Université Nationale d'Agriculture, BP 43 Kétou, République du Bénin.

***Correspondence**: dedeoutchokponhoue@gmail.com ; (D.A.T.) (+22997176987 / +27822546987)

Supplementary Materials

Table S1. Fisher R-to-Z test among sociolinguistic groups for the correlations between the number of trees owned and use value. Values in bold are the correlation coefficients between number of trees owned and use value. Values in the lower diagonal are the p-values associated to the Fisher R-to-Z test and those in the upper diagonal are the associated Z statistics.

	Adja	Aizo	Akan	Ewe	Fon	Ga-adangbe	Holli	Sahouè	Wémé
Adja		2.03	3.19	1.31	0.81	0.34	0.56	1.34	0.02
Aizo	0.04		4.85	0.98	1.55	2.41	0.78	1.63	1.21
Akan	0	0		4.61	4.29	2.93	3.25	4.42	2.73
Ewe	0.19	0.33	0		0.58	1.69	0.51	0.13	1.15
Fon	0.42	0.12	0	0.56		1.20	0.07	0.68	0.74
Ga-adangbe	0.73	0.02	0	0.09	0.23		0.87	1.71	0.28
Holli	0.58	0.27	0	0.61	0.94	0.38		0.56	0.45
Sahouè	0.18	0.43	0	0.90	0.50	0.09	0.57		0.14
Wémé	0.98	0.10	0.01	0.25	0.46	0.78	0.65	0.25	
**r**	**0.14**	**-0.2**	**0**	**-0.04**	**0.03**	**0.19**	**0.04**	**-0.06**	**0.14**

Table S2. Fisher R-to-Z test among activity categories for the correlation between the number of trees owned and use value. Values in bold are the correlation coefficients between number of trees owned and use value per age class. Values in the lower diagonal are the p-values associated to the Fisher R-to-Z test and those in the upper diagonal are the associated Z statistics.

	Young	Adult	Old
Young		3.76	3.83
Adult	0.01		2.11
Old	0	0.03	
**r**	**-0.21**	**0.04**	**0.2**

Table S3. Fisher R-to-Z test among respondents with different education levels for the correlation between the number of trees owned and use value. Values in bold are the correlation coefficients between number

of trees owned and use value for the different instruction levels. Values in the lower diagonal are the p-values associated to the Fisher R-to-Z test and those in the upper diagonal are the associated Z statistics.

	Non-educated	Literate	Primary	Secondary	>Bac
Non-educated		0.03	2.76	0.53	2.05
Literate	0.97		1.90	0.43	0.84
Primary	0.01	0.06		2.34	3.66
Secondary	0.60	0.67	0.02		2.52
>Bac	0.04	0.40	0	0.01	
**r**	**0.03**	**0.03**	**0.3**	**0.08**	**-0.21**

Table S4. Fisher R-to-Z test among respondents in various activity sectors for the correlation between the number of trees owned and use value. Values in bold are the correlation coefficients between number of trees owned and use value. Values in the lower diagonal are the p-values associated to the Fisher R-to-Z test and those in the upper diagonal are the associated Z statistics.

	Farming	Handcraft	Teaching	Trading	Traditional healing
Farming		0.01	3.06	3.62	0.36
Handcraft	0.99		1.06	1.33	0.13
Teaching	0	0.29		0.12	0.58
Trading	0	1.18	0.90		0.81
Traditional healing	0.72	0.89	0.56	0.42	
**r**	**0.1**	**0.1**	**0.34**	**0.37**	**0.13**

Table S5. Fisher R-to-Z test among respondents of religions for the correlation between the number of trees owned and use value. Values in bold are the correlation coefficients between number of trees owned and

use value. Values in the lower diagonal are the p-values associated to the Fisher R-to-Z test and those in the upper diagonal are the associated Z statistics.

	Christian	Indigenous	Muslim
Christian		1.33	198.30
Indigenous	0.18		140.30
Muslim	0	0	
**r**	**0.19**	**0.09**	**1**

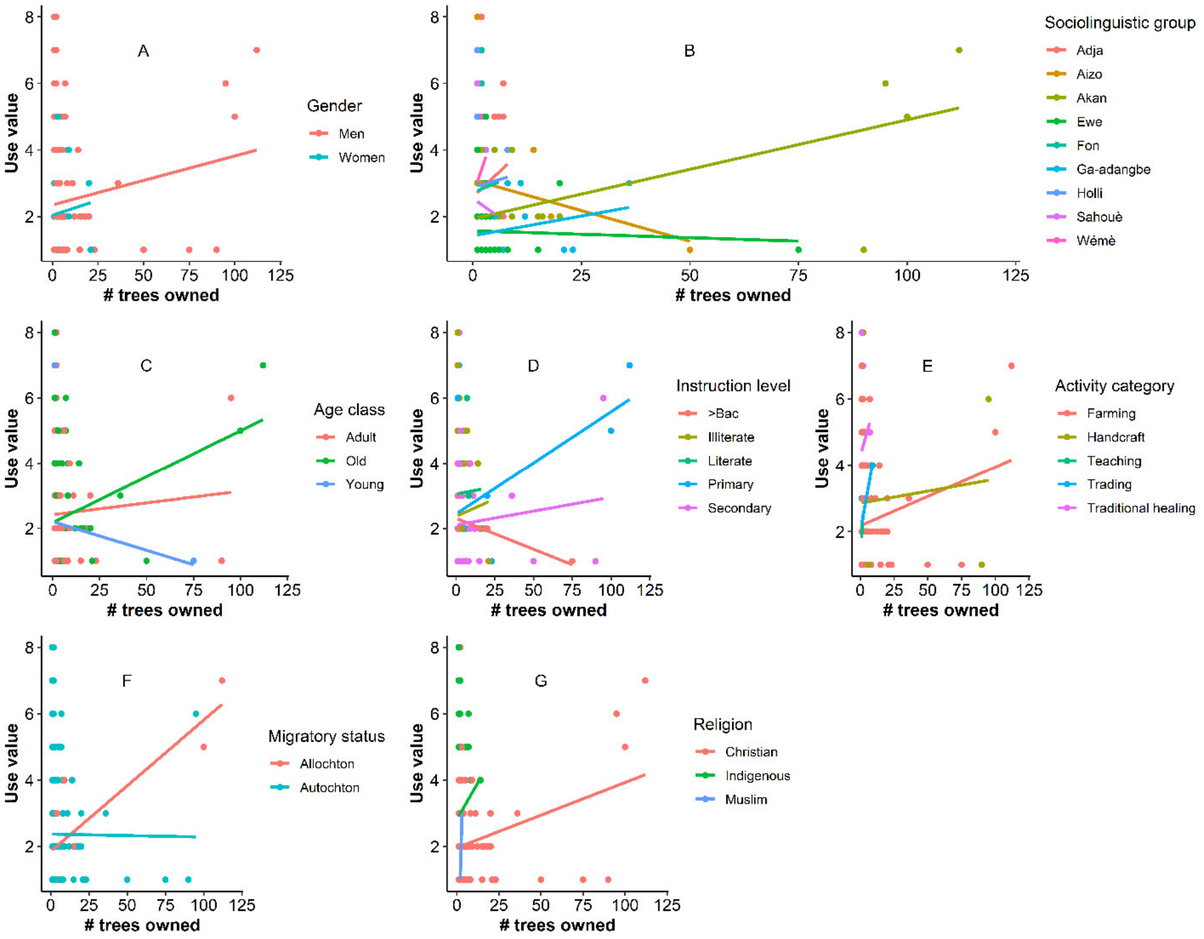

Figure S1. Regressions lines illustrating the association between use value and number of trees for attributes of different socio-demographic factors in Benin and Ghana.

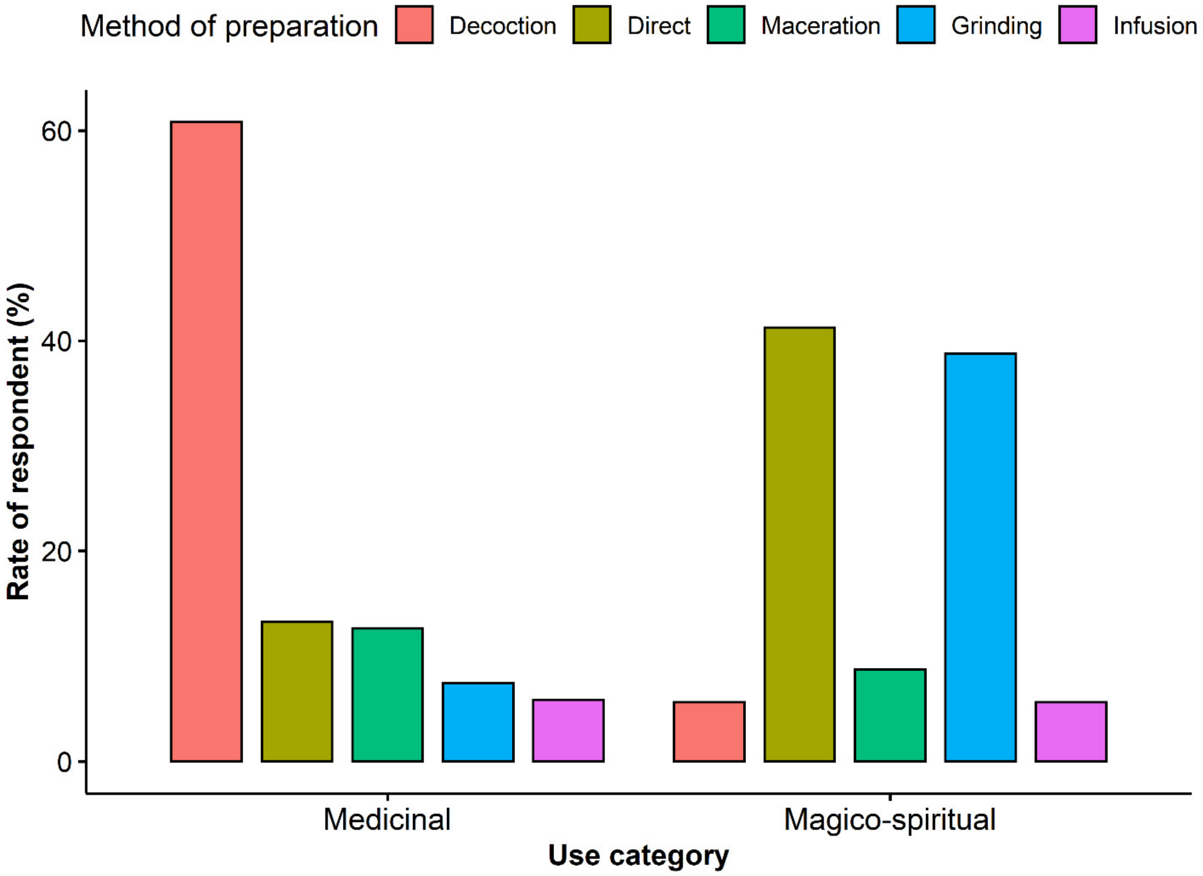

Figure S2. Relative importance of different preparation methods (all plant parts combined) for medicinal and magico-spiritual applications of the miracle plant (*Synsepalum dulcificum*) in Benin and Ghana.

Table S6. Taboos and superstitions reported on the miracle plant (*Synsepalum dulcificum*) in Benin and Ghana.

Taboos *	Concerned sociolinguistic group	Relative frequency (%)	Superstitions *	Relative frequency (%)	Concerned sociolinguistic group
It is prohibited to bring fire close to the miracle plant tress	Adja	35.28	Someone who plants the species will die or one of his parents will die before the plant starts bearing fruits	83.70	Adja
	Aizo				Aizo
It is forbidden to bring salt close to the tree	Fon	17.60	Only the elderly people plant the species	9.30	Fon
It is prohibited to bring cutlass close to the species	Holli	5.89	**Chewing the stick of the miracle plant attracts mishaps**	2.33	Holli
It is prohibited to hand-harvest the leaves	Sahouè	5.89	**Having a miracle plant tree at home attracts problems and mishaps**	2.33	Sahouè
	Wémé				Ga-adangbe

Prohibition to touch the species root	Ga-adangbe	5.89	**Having a miracle plant tree makes your problems last forever**	1.17
Prohibition to climb the tree		5.89	**The miracle plant trees are home to witches**	1.17
It is forbidden to sweep under the tree or to make a broom touch the tree		5.89	_	
Prohibition to pee on the tree		5.89	_	
**Menstruating women do not approach the tree**		5.89	_	
A pregnant woman does not approach the tree		5.89	_	

* Taboos and superstitions in bold were recorded in Ghana while those in regular font were recorded in Benin

.

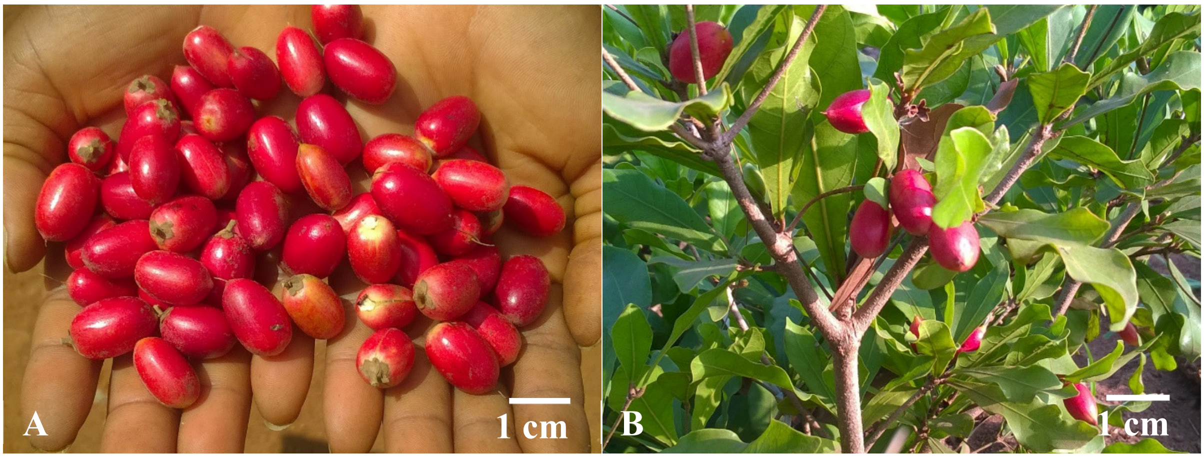

Figure S3. Plate used for the miracle plant identification by selected respondents. (**A**) The fruits (miracle fruits) and (**B**) A branch bearing fruits and other plant parts (leaves and twigs) commonly used.
